# Supplementary figures and images for: Evaluating the utility of camera traps in field studies of predation
Source: PeerJ. 2019 Feb 25;7:e6487. doi: 10.7717/peerj.6487 (PMC6394347; doi:10.7717/peerj.6487)

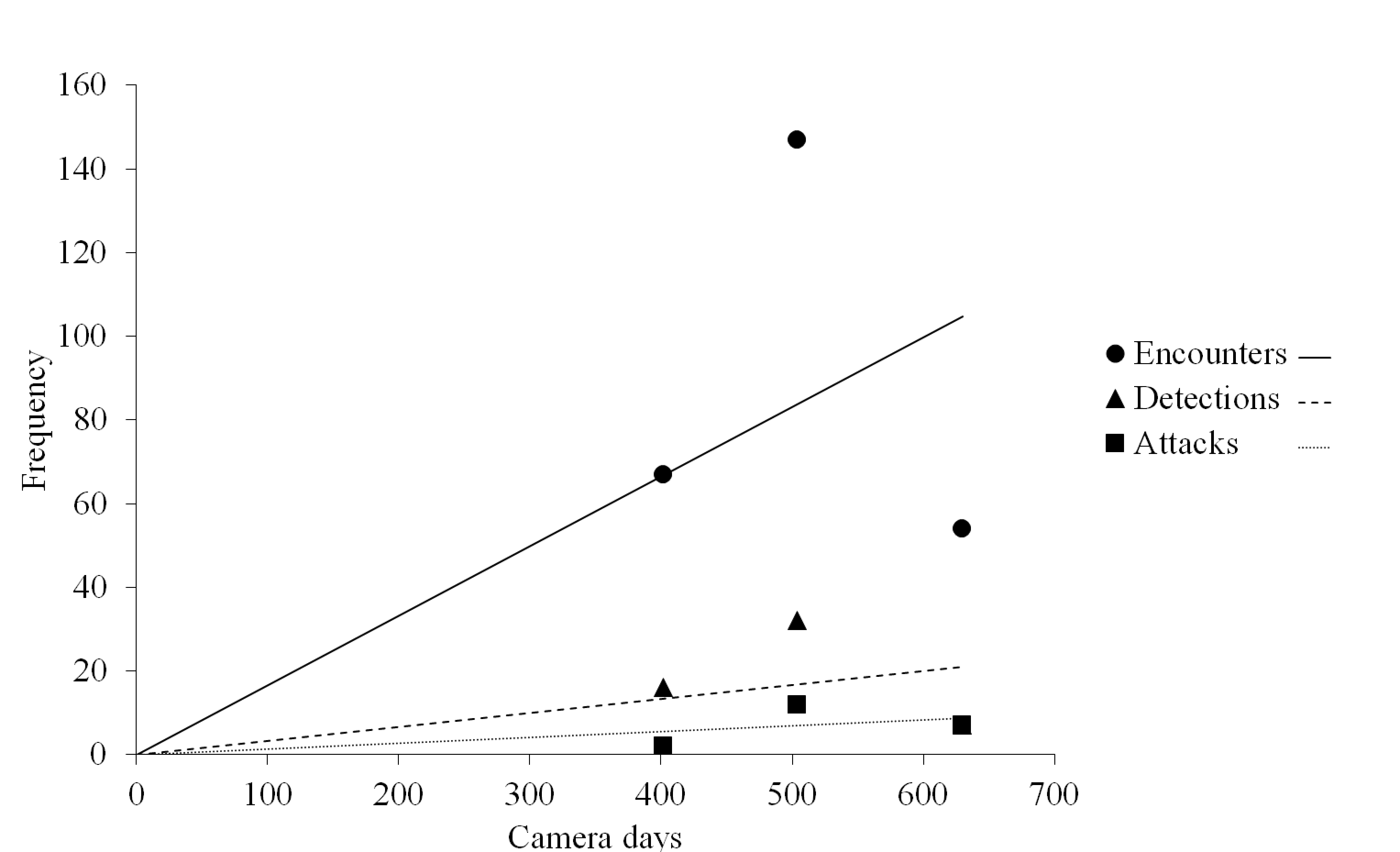

Supplement: Supplemental Information 1 — Frequency of encounters, detections, and attacks observed from camera trap videos monitoring artificial prey in Ecuador (402 camera days), Mexico (630 camera days), and North Carolina, USA (504 camera days), as a function of the number of camera days. Each point represents the frequency of either encounters, detections, or attacks from one of the field experimental locations. All regression lines were constrained to have an intercept through the origin. [file peerj-07-6487-s001.png]

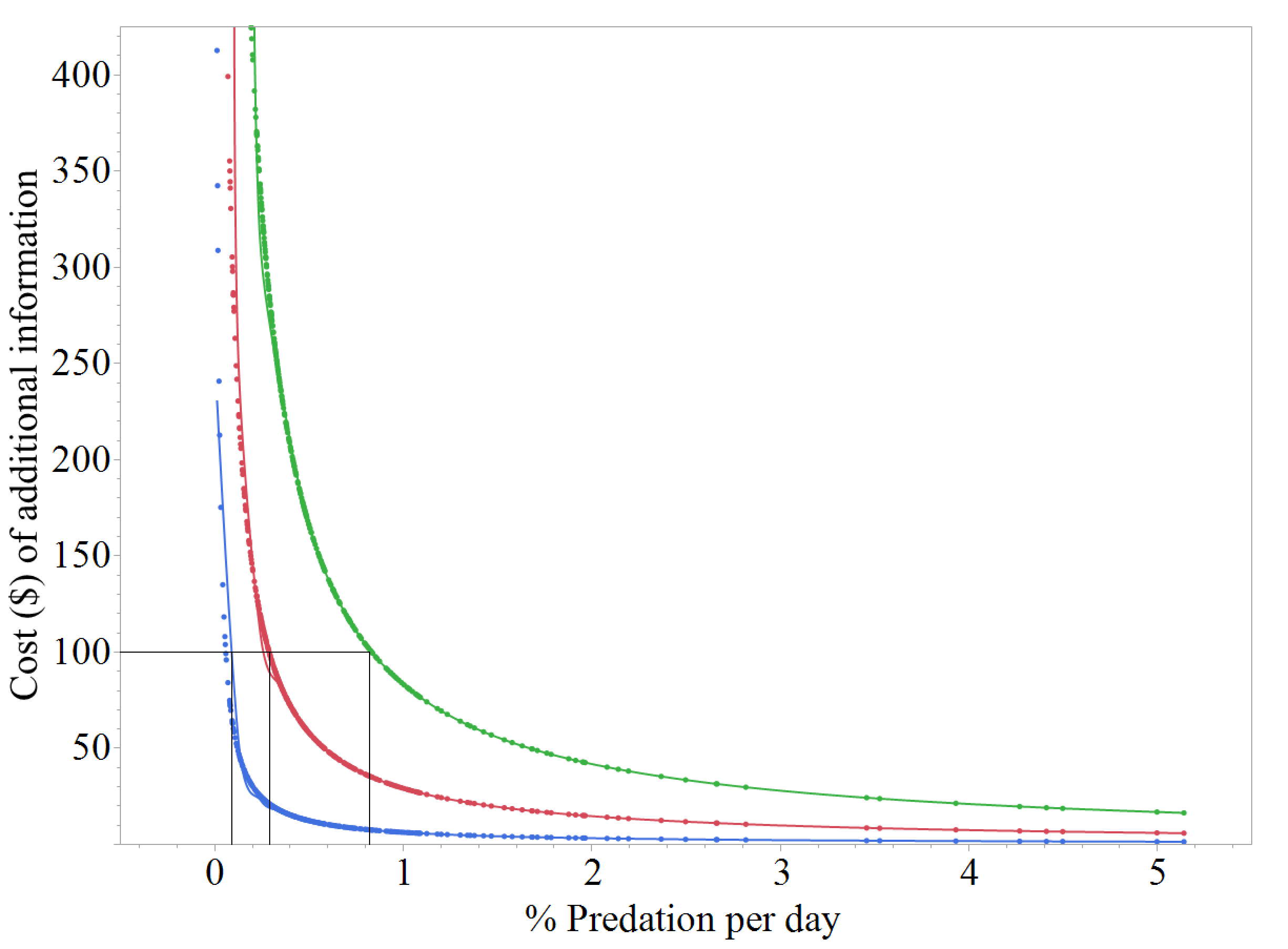

Supplement: Supplemental Information 2 — The costs of obtaining additional encounters (blue), detections (red), and attacks (green) for an artificial prey experiment that consists of 30 replicas, all monitored by cameras, that are exposed to natural predators for 12 days as a function of the predator activity level (% predation per day as a proxy) estimated from each of 424 artificial prey studies. Differences in the rates of encounters, detections, and attacks from other studies were assumed to be proportional to the rates of encounters, detections, and attacks estimated from this study. Black lines show the minimum predator activity level that would be necessary for the purchase of one additional $100 camera to capture an additional encounter, detection, or attack. [file peerj-07-6487-s002.png]
