# Supplementary material for: Evaluating the utility of camera traps in field studies of predation
Source: PeerJ. 2019 Feb 25;7:e6487. doi: 10.7717/peerj.6487 (PMC6394347; doi:10.7717/peerj.6487)
Supplement: Supplemental Information 3 — Species in bold were considered to be potential predators of snakes in analyses. Nomenclature follows Ridgely & Greenfield (2001), Wilson & Reeder (2005), Peterson (2010), and Vallely & Dyer (2018). [file peerj-07-6487-s003.docx]

| Ecuador |  | Common Name | Scientific Name |
| --- | --- | --- | --- |
|  | Snake  Bird | South American coral snake  Ornate coral snake  Worm-eating coral snake  Amazonian coral snake  Aquatic coral snake | *Micrurus lemniscatus*  *Micrurus ornatissimus*  *Micrurus ortoni*  *Micrurus spixii*  *Micrurus surinamensis* |
|  |  | **Brown nunlet** | ***Nonnula brunnea*** |
|  |  | **Slate-colored hawk** | ***Buteogallus schistaceus*** |
|  |  | **Gray-winged trumpeter** | ***Psophia crepitans*** |
|  |  | Sapphire quail-dove | *Geotrygon saphirina* |
|  |  | Ruddy quail-dove | *Geotrygon montana* |
|  |  | Great tinamou | *Tinamus major* |
|  |  | White-throated tinamou | *Tinamus guttatus* |
|  |  | Variegated tinamou | *Crypturellus variegatus* |
|  |  | Undulated tinamou | *Crypturellus undulates* |
|  |  | Cinereous tinamou | *Crypturellus cinereus* |
|  |  | Gray-fronted dove | *Leptotila rufaxilla* |
|  |  | Rufous-capped antthrush | *Formicarius colma* |
|  |  | White-necked thrush | *Turdus albicollis* |
|  | Mammal |  |  |
|  |  | **Nine-banded armadillo** | ***Dasypus novemcinctus*** |
|  |  | **Giant armadillo** | ***Priodontes maximus*** |
|  |  | **Ocelot** | ***Leopardus pardalis*** |
|  |  | **White-lipped peccary** | ***Tayassu pacari*** |
|  |  | **Collared peccary** | ***Peccari tajacu*** |
|  |  | South American red brocket deer | *Mazama americana* |
|  |  | Lowland paca | *Cuniculus paca* |
|  |  | Black agouti | *Dasyprocta fuliginosa* |
|  |  | Unidentified small rodents | *Rodentia* spp. |
|  | Reptile |  |  |
|  |  | Forest whiptail | *Kentropyx pelviceps* |
| Mexico |  | Common Name | Scientific Name |
|  | Snake | Variable coral snake  Elegant coral snake  Variegated false coral snake | *Micrurus diastema*  *Micrurus elegans*  *Pliocercus elapoides* |
|  | Bird |  |  |
|  |  | **Lesson’s motmot** | ***Momotus lessonii*** |
|  |  | Orange-billed sparrow | *Arremon aurantiirostris* |
|  |  | Spotted wood-quail | *Odontophorus guttatus* |
|  |  | Slaty-breasted tinamou | *Crypturellus boucardi* |
|  |  | Great tinamou | *Tinamus major* |
|  |  | Little tinamou | *Crypturellus soui* |
|  |  | Ruddy quail-dove | *Geotrygon montana* |
|  |  | Gray-headed dove | *Leptotila plumbeiceps* |
|  |  | White-bellied wren | *Uropsila leucogastra* |
|  |  | Clay-colored thrush | *Turdus grayi* |
|  |  | Plain chachalaca | *Ortalis vetula* |
|  |  | Hummingbirds | *Trochilidae* sp. |
|  | Mammal |  |  |
|  |  | **Tayra** | ***Eira barbara*** |
|  |  | **Ocelot** | ***Leopardus pardalis*** |
|  |  | **Jaguarundi** | ***Puma yagouaroundi*** |
|  |  | **Common raccoon** | ***Procyon lotor*** |
|  |  | **White-nosed coati** | ***Nasua narica*** |
|  |  | **Hooded skunk** | ***Mephitis macroura*** |
|  |  | **Gray fox** | ***Urocyon cinereoargenteus*** |
|  |  | **Nine-banded armadillo** | ***Dasypus novemcinctus*** |
|  |  | **Common opossum** | ***Didelphis marsupialis*** |
|  |  | Lowland paca | *Cuniculus paca* |
|  |  | Central American agouti | *Dasyprocta punctata* |
|  |  | Squirrels | *Sciurus* sp. |
|  |  | Unidentified small rodents | *Rodentia* spp. |
|  | Reptile |  |  |
|  |  | Blue spiny lizard | *Sceloporus serrifer* |
|  |  | Jungle runners | *Ameiva* sp. |
| North Carolina |  | Common Name | Scientific Name |
|  | Snake | Eastern coral snake | *Micrurus fulvius* |
|  | Bird |  |  |
|  |  | **American crow** | ***Corvus brachyrhynchos*** |
|  |  | **Wild turkey** | ***Meleagris gallopavo*** |
|  |  | Hermit thrush | *Catharus guttatus* |
|  |  | Northern cardinal | *Cardinalis cardinalis* |
|  | Mammal |  |  |
|  |  | **Gray fox** | ***Urocyon cinereoargenteus*** |
|  |  | **Virginia opossum** | ***Didelphis virginiana*** |
|  |  | **Common raccoon** | ***Procyon lotor*** |
|  |  | **Black bear** | ***Ursus americanus*** |
|  |  | Fox squirrel | *Sciurus niger* |
|  |  | Eastern gray squirrel | *Sciurus carolinensis* |
|  |  | White-tailed deer | *Odocoileus virginianus* |
|  |  | Eastern cottontail | *Sylvilagus floridanus* |
