# Supplementary material for: Evaluating the utility of camera traps in field studies of predation
Source: PeerJ. 2019 Feb 25;7:e6487. doi: 10.7717/peerj.6487 (PMC6394347; doi:10.7717/peerj.6487)
Supplement: Supplemental Information 4 — Equipment and supply costs represent the actual costs for listed items. Personnel wages were assumed to be equivalent for all experiments. For Ecuador and Mexico, travel and lodging costs represent the actual costs that were expended to travel to and from field sites. For North Carolina, a standard government travel rate was used to estimate travel costs. [file peerj-07-6487-s004.docx]

| Ecuador | **Without Cameras** | **Cost ($)** | **Added Cost of Cameras** | **Cost ($)** |
| --- | --- | --- | --- | --- |
| Equipment and Supplies | 20 kg Sculpey III Clay | 478 | 21 Spypoint Force 10 | 1,890 |
|  | 1350 pieces of 20 gauge 18’’ stem wire | 95 | 10 Scout Guard SG560V-31B | 900 |
|  | Polymer clay extruder | 110 | 1 ANNKE C303 | 80 |
|  | Pliers | 5 | 32 SD Cards (32 GB) | 320 |
|  |  |  | 214 AA batteries | 64 |
| Personnel ($15/hour) | Replicas checked every 2 days | 840 | Extra baggage fee  Data processing (9 hours) | 150  135 |
|  |  |  |  |  |
| Travel | Airfares | 1,124 | Total | **$3,539** |
|  | Ground Transportation | 120 |  |  |
| Lodging | 14 nights | 644 |  |  |
|  | Total | **$3,416** | **Total for Experiment** | **$6,955** |
|  |  |  |  |  |
| Mexico | **Without Cameras** | **Cost ($)** | **Added Cost of Cameras** | **Cost ($)** |
| Equipment and Supplies | 24 kg Sculpey III Clay | 575 | 21 Spypoint Force 10 | 1,890 |
|  | 1400 pieces of 20 gauge 18’’ stem wire | 98 | 1 ANNKE C303 | 80 |
|  | Polymer clay extruder | 110 | 22 SD Cards (32 GB) | 220 |
|  | Pliers | 5 | 134 AA batteries | 42 |
| Personnel ($15/hour) | Replicas checked every 6 days | 600 | Extra baggage fee  Data processing (12 hours) | 150  180 |
|  |  |  |  |  |
| Travel | Airfare | 555 | Total | **$2,562** |
|  | Ground Transportation | 250 |  |  |
| Lodging | 30 days | 100 |  |  |
|  | Total | **$2,293** | **Total for Experiment** | **$4,855** |
|  |  |  |  |  |
| North Carolina, USA | **Without Cameras** | **Cost ($)** | **Added Cost of Cameras** | **Cost ($)** |
| Equipment and Supplies | 12 kg Sculpey III Clay | 287 | 21 Spypoint Force 10 | 1,890 |
|  | 600 pieces of 20 gauge 18’’ stem wire | 42 | 1 ANNKE C303 | 80 |
|  | Polymer clay extruder | 110 | 1 Bestguarder DTC-880V | 90 |
|  | Pliers | 5 | 23 SD Cards (32 GB) | 230 |
|  | 200 6-in 2 gauge nails | 80 | 142 AA batteries | 44 |
|  | 200-g fishing line | 15 | Data processing (11 hours) | 165 |
|  |  |  |  |  |
| Personnel ($15/hour) | Replicas picked up after 28 days | 240 |  |  |
|  |  |  | Total | **$2,499** |
| Travel ($0.54/mile) | Two 400-mile trips | 432 |  |  |
|  | Total | **$1,211** | **Total for Experiment** | **$3,710** |
|  |  |  |  |  |
