# Supplementary material for: Evaluating the utility of camera traps in field studies of predation
Source: PeerJ. 2019 Feb 25;7:e6487. doi: 10.7717/peerj.6487 (PMC6394347; doi:10.7717/peerj.6487)
Supplement: Supplemental Information 5 — Museums that contributed specimens that were used to aid the construction of artificial snake replicas. [file peerj-07-6487-s005.docx]

| Inst. Code | Institution Name |
| --- | --- |
| AMNH | American Museum of Natural History, New York City, NY |
| AUMNH | Auburn University Natural History Museum and Learning Center, Auburn, AL |
| BRTC | Texas Cooperative Wildlife Collection, now Biodiversity Research and Teaching Collections, Texas A&M University, College Station, TX |
| BYU | Monte L. Bean Life Science Museum, Brigham Young University, Provo, UT |
| CM | Carnegie Museum of Natural History, Philadelphia, PA |
| FLMNH | Florida Museum of Natural History, Gainesville, FL |
| FMNH | Field Museum of Natural History, Chicago, IL |
| GSU | Georgia State University, Statesboro, GA |
| INHS | Illinois Natural History Survey, University of Illinois, Champaign, IL |
| LSU | Louisiana Museum of Natural History, Louisiana State University, Baton Rouge, LA |
| MISS | Mississippi Museum of Natural History, Jackson, MS |
| MPM | Milwaukee Public Museum, Milwaukee, WI |
| NCSM | North Carolina State Museum of Natural Sciences, now the North Carolina Museum of Natural Sciences, Raleigh, NC |
| OMNH | Sam Noble Oklahoma Museum, University of Oklahoma, Norman, OK |
| QCAZ | Zoology Museum, Pontifical Catholic University of Ecuador, Quito, Ecuador |
| UMNH | Utah Museum of Natural History, University of Utah, Salt Lake City, UT |
| USNM | Smithsonian National Museum of Natural History, Washington, D.C. |
| UTA | University of Texas at Arlington Amphibian and Reptile Diversity Research Center, Arlington, TX |
| UTEP | The Centennial Museum, University of Texas at El Paso, El Paso, TX |
