# Supplementary material for: Evaluating the utility of camera traps in field studies of predation
Source: PeerJ. 2019 Feb 25;7:e6487. doi: 10.7717/peerj.6487 (PMC6394347; doi:10.7717/peerj.6487)
Supplement: Supplemental Information 19 — References for all of the studies from the literature survey that were used to estimate the cost of camera traps. [file peerj-07-6487-s019.docx]

**References from Literature Survey**

Ackerman JT, Blackmer AL, and Eadie JM. 2004. Is predation on waterfowl nests density dependent?: tests at three spatial scales. *Oikos* 107:128–140 DOI:10.1111/j.0030-1299. 2004.13226.x.

Albrecht T, Hořák D, Kreisinger J, Weidinger K, Klvaňa P, and Michot TC. 2006. Factors determining pochard nest predation along a wetland gradient. *Journal of Wildlife Management* 70:784–791 DOI:10.2193/0022-541X(2006)70[784:FDPNPA]2.0.CO;2.

Almario BS, Marra PP, Gates JE, and Mitchell L. 2009. Effects of prescribed fire on depredation rates of natural and artificial seaside sparrow nests. *The Wilson Journal of Ornithology* 121:770-777 DOI:10.1676/07-095.1.

Almeida MV, Lucundo AS, Costa TVV, Paula HMG. 2013. Predation on artificial nests by marmosets of the genus *Callithrix* (primates, platyrrhini) in a cerrado fragment in southeastern Brazil. *Biotemas* 26:203-207 DOI:10.5007/2175-7925.2013v26n1p203.

Amézquita A, Castro L, Arias M, González M, and Esquivel C. 2013. Field but not lab paradigms support generalization by predators of aposematic polymorphic prey: the  *Oophaga histrionica* complex. *Evolutionary Ecology* 27:769-782 DOI:10.1007/s10682- 013-9635-1.

Andersen DC, and MacMahon JA. 1986. An assessment of ground-nest depredation in a catastrophically disturbed region, Mount St. Helens, Washington. *The Auk* 103:622-626.

Anderson CJ, Hostetler ME, Sieving KE, and Johnson SA. 2016. Predation of artificial nests by introduced rhesus macaques (*Macaca mulatta*) in Florida, USA. *Biological Invasions* 18: 2783-2789 DOI:10.1007/s10530-016-1195-1.

Andersson M, and Wiklund CG. 1978. Clumping versus spacing out: experiments on nest predation in fieldfares (*Turdus pilaris*). *Animal Behavior* 26:1207-1212 DOI:10.1016/000 3-3472(78)90110-0.

Andren H, and Angelstam P. 1988. Elevated predation rates as an edge effect in habitat islands: experimental evidence. *Ecology* 69:544-547 DOI:10.2307/1940455.

Andrén C, and Nilson G. 1981. Reproductive success and risk of predation in normal and melanistic colour morphs of the adder, *Vipera berus*. *Biological Journal of the Linnean Society* 15:235-246 DOI:10.1111/j.1095-8312.1981.tb00761.x.

Andrén H, Angelstam P, Lindström E, and Widén P. 1985. Differences in predation pressure in relation to habitat fragmentation: an experiment. *Oikos* 45:273-277 DOI:10.2307/35657 14.

Arango-Vélez N, and Kattan GH. 1997. Effects of forest fragmentation on experimental nest predation in Andean cloud forest. *Biological Conservation* 81:137-143 DOI:10.1016/S 0006-3207(96)00138-3.

Ardizzone CD, and Norment CJ. 1999. Experimental analysis of nest predation in a New York grassland: effects of habitat and nest distributions. *Studies in Avian Biology* 19:122-127.

Arias M, Poul Y, Chouteau M, Boisseau R, Rosser N, Théry M, and Llaurens V. 2016. Crossing fitness valleys: empirical estimation of a fitness landscape associated with polymorphic mimicry. *Proceedings of the Royal Society of London B: Biological Sciences* 283:201603 91 DOI:10.1098/rspb.2016.0391.

Baines D. 1990. The roles of predation, food and agricultural practice in determining the breeding success of the lapwing (*Vanellus vanellus*) on upland grasslands. *Journal of Animal Ecology* 59:915-929 DOI:10.2307/5022.

Báldi A, and Batáry P. 2005. Nest predation in European reedbeds: different losses in edges but similar losses in interiors. *Folia Zoologica* 54:285-292.

Ball JR, Bayne EM, and Machtans CS. 2009. Video identification of boreal forest songbird nest predation and discordance with artificial nest studies. In: Rich TD, Armizmendia C, Demarest D, and Thompson C, eds. *Tundra to tropics: connecting birds, habitats and people.* Proceedings of the Fourth International Partners in Flight Conference, 13-16 February 2008, McAllen, TX, 37-44.

Batáry P, Winkler H, and Báldi A. 2004. Experiments with artificial nests on predation in reed habitats. *Journal of Ornithology* 145:59-63 DOI:10.1007/s10336-003-0010-9.

Batáry P, Fronczek S, Normann C, Scherber C, and Tscharntke T. 2014. How do edge effect and tree species diversity change bird diversity and avian nest survival in Germany’s largest deciduous forest? *Forest Ecology and Management* 319:44-59 DOI:10.1016/j.foreco. 2014.02.004.

Bayne EM, and Hobson KA. 1999. Do clay eggs attract predators to artificial nests? *Journal of Field Ornithology* 70:1-7.

Bayne EM, and Hobson KA. 2002. Effects of red squirrel (*Tamiasciurus hudsonicus*) removal on survival of artificial songbird nests in boreal forest fragments. *American Midland Naturalist* 147:72-79 DOI:10.1674/0003-0031(2002)147[0072:EORSTH]2.0.CO;2.

Bayne EM, Hobson KA, and Fargey P. 1997. Predation on artificial nests in relation to forest type: contrasting the use of quail and plasticine eggs. *Ecography* 20:233-239 DOI: 10.1111/j.1600-0587.1997.tb00366.x.

Belthoff JR. 2005. Using artificial nests to study nest predation in birds. *The American Biology Teacher* 67:105-110 DOI:10.1662/0002-7685(2005)067[0105:UANTSN]2.0.CO;2.

Berg A. 1996. Predation on artificial, solitary, and aggregated wader nests on farmland.  *Oecologia* 107:343-346 DOI:10.1007/BF00328450.

Bergin TM, Best LB, and Freemark KE. 1997. An experimental study of predation on artificial nests in roadsides adjacent to agricultural habitats in Iowa. *The Wilson Bulletin* 109:437- 448.

Bergin TM, Best LB, Freemark KE, and Koehler KJ. 2000. Effects of landscape structure on nest predation in roadsides of a midwestern agroecosystem: a multiscale analysis. *Landscape Ecology* 15:131-143.

Berry L. 2002. Predation rates of artificial nests in the edge and interior of a southern Victorian forest. *Wildlife Research* 29:341-345 DOI:10.1071/WR01022.

Bety J, Gauthier G, Giroux JF, and Korpimäki E. 2001. Are goose nesting success and lemming cycles linked? Interplay between nest density and predators. *Oikos* 93:388-400 DOI:10. 1034/j.1600-0706.2001.930304.x.

Bittner TD. 2003. Polymorphic clay models of *Thamnophis sirtalis* suggest patterns of avian predation. *Ohio Journal of Science* 103:62-66.

Blamires SJ, Spencer RJ, King P, and Thompson MB. 2005. Population parameters and life-table analysis of two coexisting freshwater turtles: are the Bellinger River turtle populations threatened? *Wildlife Research* 32:339-347 DOI:10.1071/WR04083.

Boag DA, Reebs SG, and Schroeder MA. 1984. Egg loss among spruce grouse inhabiting lodgepole pine forests. *Canadian Journal of Zoology* 62:1034-1037 DOI:10.1139/z84- 147.

Bobo KS, and Waltert M. 2011. Artificial bird nest predation along a forest conversion gradient in Cameroon. *Ecotropica* 17:21-29.

Borgmann KL, and Rodewald AD. 2004. Nest predation in an urbanizing landscape: the role of exotic shrubs. *Ecological Applications* 14:1757-1765 DOI:10.1890/03-5129.

Borgo JS, and Conover MR. 2015. Spatio-temporal patterns in the depredation of waterfowl nests and simulated nests in the prairie pothole region, USA. *Waterbirds* 38:133-142 DOI:10.1675/063.038.0202.

Boulet M, Darveau M, and Bélanger L. 2003. Nest predation and breeding activity of songbirds in riparian and nonriparian black spruce strips of central Quebec. *Canadian Journal of Forest Research* 33:922-930 DOI:10.1139/X03-029.

Boulton RL, and Clarke MF. 2003. Do yellow-faced honeyeater (*Lichenostomus chrysops*) nests experience higher predation at forest edges? *Wildlife Research* 30:119-125 DOI:10.1071/ WR02055.

Boulton RL, Cassey P, Schipper C, and Clarke MF. 2003. Nest site selection by yellow-faced honeyeaters *Lichenostomus chrysops*. *Journal of Avian Biology* 34:267-274 DOI:10. 1034/j.1600-048X.2003.03062.x.

Bowen DE, Robel RJ, and Watt PG. 1976. Habitat and investigators influence artificial ground nest losses: Kansas. *Transactions of the Kansas Academy of Science* 79:141-147 DOI: 10.2307/3627293.

Boyle WA. 2008. Can variation in risk of nest predation explain altitudinal migration in tropical birds? *Oecologia* 155:397-403 DOI:10.1007/s00442-007-0897-6.

Brand LA, and George TL. 2000. Predation risks for nesting birds in fragmented coast redwood forest. *The Journal of Wildlife Management* 64:42-51 DOI:10.2307/3802973.

Brennan PLR. 2010. Clutch predation in great tinamous *Tinamus major* and implications for the evolution of egg color. *Journal of Avian Biology* 41:419-426 DOI:10.1111/j.1600-048X. 2010.04999.x.

Brodie ED. 1993. Differential avoidance of coral snake banded patterns by free-ranging avian predators in Costa Rica. *Evolution* 47:227-235 DOI:10.1111/j.1558- 5646.1993.tb01212.x.

Brodie ED, and Janzen FJ. 1995. Experimental studies of coral snake mimicry: generalized avoidance of ringed snake patterns by free-ranging avian predators. *Functional Ecology* 9:186-190 DOI:10.2307/2390563.

Buasso CM, Leynaud GC, and Cruz FB. 2006. Predation on snakes of Argentina: effects of coloration and ring pattern on coral and false coral snakes. *Studies on Neotropical Fauna and Environment* 41:183–188 DOI:10.1080/01650520600630725.

Buehler R, Bosco L, Arlettaz R, and Jacot A. 2017. Nest site preferences of the woodlark (*Lullula arborea*) and its association with artificial nest predation. *Acta Oecologica* 78: 41-46 DOI:10.1016/j.actao.2016.12.004.

Buler JL, and Hamilton RB. 2000. Predation of natural and artificial nests in a southern pine forest. *The Auk* 117:739-747 DOI:10.1642/0004-8038(2000)117[0739:PONAAN]2.0. CO;2.

Burger LD, Burger Jr LW, and Faaborg J. 1994. Effects of prairie fragmentation on predation on artificial nests. *The Journal of Wildlife Management* 58:249-254 DOI:10.2307/3809387.

Burkey TV. 1993. Edge effects in seed and egg predation at two neotropical rainforest sites.  *Biological Conservation* 66:139-143 DOI:10.1016/0006-3207(93)90145-Q.

Butler MA, and Rotella JJ. 1998. Validity of using artificial nests to assess duck-nest success.  *The Journal of Wildlife Management* 62:163-171 DOI:10.2307/3802274.

Buzuleciu SA, Crane DP, and Parker SL. 2016. Scent of disinterred soil as an olfactory cue used by raccoons to locate nests of diamond-backed terrapins (*Malaclemys terrapin*).  *Herpetological Conservation and Biology* 11:539-551.

Byrkjedal I. 1980. Nest predation in relation to snow-cover: a possible factor influencing the start of breeding in shorebirds. *Ornis Scandinavica* 11:249-252 DOI:10.2307/3676131.

Capula M, Luiselli L, and Capanna E. 1997. The blue-spotted morph of the slow worm, *Anguis fragilis*: colour polymorphism and predation risks. *Italian Journal of Zoology* 64:147-153 DOI:10.1080/11250009709356188.

Carignan V, and Villard MA. 2002. Effects of variations in micro-mammal abundance on artificial nest predation in conifer plantations and adjoining deciduous forests. *Forest Ecology and Management* 157:255-265 DOI:10.1016/S0378-1127(00)00650-2.

Carpio AJ, Tortosa FS, and Barrio IC. 2015. Rabbit abundance influences predation on bird nests in Mediterranean olive orchards. *Acta Ornithologica* 50:171-179 DOI:10.3161/00016454 AO2015.50.2.005.

Carpio AJ, Hillström L, and Tortosa FS. 2016. Effects of wild boar predation on nests of wading birds in various Swedish habitats. *European Journal of Wildlife Research* 62:423-430 DOI:10.1007/s10344-016-1016-y.

Castilla AM. 1995. Intensive predation of artificial Audouin’s gull nests by the yellow-legged gull in the Columbretes Islands, Spain. *Colonial Waterbirds* 18:226-230 DOI:10.2307/ 1521487.

Castilla AM, and Labra A. 1998. Predation and spatial distribution of the lizard *Podarcis hispanica atrata*: an experimental approach. *Acta Oecologica* 19:107-114 DOI:10.1016/ S1146-609X(98)80014-3.

Castilla AM, Gosá A, Galán P, and Pérez-Mellado V. 1999. Green tails in lizards of the genus  *Podarcis*: do they influence the intensity of predation? *Herpetologica* 55:530-537.

Castro-Caro JC, Carpio AJ, and Tortosa FS. 2014. Herbaceous ground cover reduces nest predation in olive groves. *Bird Study* 61:537-543 DOI:10.1080/00063657.2014.961894.

Cervantes-Cornihs E, Zuria I, and Castellanos I. 2009. Artificial nest predation in hedgerows of an agro-urban system in Hidalgo, Mexico. *Interciencia* 34:777-783.

Chamberlain DE, Hatchwell BJ, and Perrins CM. 1995. Spaced out nests and predators: an experiment to test the effects of habitat structure. *Journal of Avian Biology* 26:346-349 DOI:10.2307/3677052.

Chiarello AG, Srbek-Araujo AC, Del Duque Jr HJ, and Coelho ER. 2008. Ground nest predation might not be higher along edges of Neotropical forest remnants surrounded by pastures: evidence from the Brazilian Atlantic forest. *Biodiversity and Conservation* 17:3209-3221 DOI:10.1007/s10531-008-9422-4.

Chibowski P, Brzeziński M, and Jedlikowski J. 2015. Predation on artificial nests imitating the broods of two rallid species: the influence of habitat features. *Polish Journal of Ecology* 63:573-584 DOI:10.3161/15052249PJE2015.63.4.009.

Chouteau M, and Angers B. 2011. The role of predators in maintaining the geographic organization of aposematic signals. *The American Naturalist* 178:810-817 DOI:1086/ 662667.

Chouteau M, and Angers B. 2012. Wright’s shifting balance theory and the diversification of aposematic signals. *PLoS ONE* 7:e34028 DOI:10.1371/journal.pone.0034028.

Cimadom A, Ulloa A, Meidl P, Zöttl M, Zöttl E, Fessl B, Nemeth E, Dvorak M, Cunninghame F, and Tebbich S. 2014. Invasive parasites, habitat change and heavy rainfall reduce breeding success in Darwin’s Finches. *PLoS ONE* 9:e107518 DOI:10.1371/journal.pone. 0107518.

Clark RG, and Wobeser BK. 1997. Making sense of scents: effects of odour on survival of simulated duck nests. *Journal of Avian Biology* 28:31-37 DOI:10.2307/3677091.

Clawson MR, and Rotella JJ. 1998. Success of artificial nests in CRP fields, native vegetation, and field borders in southwestern Montana. *Journal of Field Ornithology* 69:180-191.

Colombelli-Négrel D, and Kleindorfer S. 2009. Nest height, nest concealment, and predator type predict nest predation in superb fairy-wrens (*Malurus cyaneus*). *Ecological Research* 24: 921-928 DOI:10.1007/s11284-008-0569-y.

Conner LM, and Perkins MW. 2003. Nest predator use of food plots within a forest matrix: an experiment using artificial nests. *Forest Ecology and Management* 179:223-229 DOI:10. 1016/S0378-1127(02)00516-9.

Cooney SJN, and Watson DM. 2008. An experimental approach to understanding the use of mistletoe as a nest substrate for birds: nest predation. *Wildlife Research* 35:65-71 DOI:10. 1071/WR06144.

Cooper SM, and Ginnett TF. 2000. Potential effects of supplemental feeding of deer on nest predation. *Wildlife Society Bulletin* 28:660-666.

Cotterill SE, and Hannon SJ. 1999. No evidence of short-term effects of clear-cutting on artificial nest predation in boreal mixedwood forests. *Canadian Journal of Forest Research* 29:1900-1910 DOI:10.1139/x99-153.

Cuthbert R, and Hilton G. 2004. Introduced house mice *Mus musculus*: a significant predator of threatened and endemic birds on Gough Island, South Atlantic Ocean? *Biological Conservation* 117:483-489 DOI:10.1016/j.biocon.2003.08.007.

Daly BG, Dickman CR, and Crowther MS. 2008. Causes of habitat divergence in two species of agamid lizards in arid central Australia. *Ecology* 89:65-76 DOI:10.1890/06-1991.1.

Darveau M, Belanger L, Huot J, Melancon E, and DeBellefeuille S. 1997. Forestry practices and the risk of bird nest predation in a boreal coniferous forest. *Ecological Applications* 7:572 -580 DOI:10.1890/1051-0761(1997)007[0572:FPATRO]2.0.CO;2.

Davison WB, and Bollinger E. 2000. Predation rates on real and artificial nests of grassland birds. *The Auk* 117:147-153 DOI:10.1642/0004-8038(2000)117[0147:PRORAA]2.0.CO; 2.

De Santo TL, and Willson MF. 2001. Predator abundance and predation of artificial nests in natural and anthropogenic coniferous edges in southeast Alaska. *Journal of Field Ornithology* 72:136-149 DOI:10.1648/0273-8570-72.1.136.

DeGraaf RM. 1995. Nest predation rates in managed and reserved extensive northern hardwood forests. *Forest Ecology and Management* 79:227-234 DOI:10.1016/0378-1127(95)0359 4-X.

DeGraaf RM, and Angelstam P. 1993. Effects of timber size-class on predation of artificial nests in extensive forest. *Forest Ecology and Management* 61:127-136 DOI:10.1016/0378-112 7(93)90194-R.

DeGraaf RM, Maier TJ, and Fuller TK. 1999. Predation of small eggs in artificial nests: effects of nest position, edge, and potential predator abundance in extensive forest. *The Wilson Bulletin* 111:236-242.

DeGroote LW, Ober HK, and McDonough CM. 2013. An evaluation of the nine-banded armadillo as predators of gopher tortoise and northern bobwhite quail nests in Florida.  *The American Midland Naturalist* 169:74-85 DOI:10.1674/0003-0031-169.1.74.

Delgado García JD, Arévalo JR, and Fernández-Palacios JM. 2005. Patterns of artificial avian nest predation by introduced rats in a fragmented laurel forest (Tenerife, Canary Islands).  *Journal of Natural History* 38:2661-2669 DOI:10.1080/00222930500104427.

Dell’Aglio DD, Stevens M, and Jiggins CD. 2016. Avoidance of an aposematically coloured butterfly by wild birds in a tropical forest. *Ecological Entomology* 41:627-632 DOI:10. 1111/een.12335.

Dias RI, Castilho L, Macedo RH. 2010. Experimental evidence that sexual displays are costly for nest survival. *Ethology* 116:1011-1019 DOI:10.1111/j.1439-0310.2010.01817.x.

Dinkins JB, Conover MR, and Mabray ST. 2013. Do artificial nests simulate nest success of greater sage-grouse? *Human-Wildlife Interactions* 7:299-312.

Djomo Nana E, Sedláček O, Vokurková J, and Hořák D. 2014. Nest position and type affect predation rates of artificial avian nests in the tropical lowland forest on Mount Cameroon.  *Ostrich* 85:93-96 DOI:10.2989/00306525.2014.900830.

Dodonov P, Paneczko IT, and Telles M. 2017. Edge, height and visibility effects on nest predation by birds and mammals in the Brazilian *cerrado*. *Acta Oecologica* 83:56-64 DOI:10.1016/j.actao.2017.06.010.

Donalty SM, and Henke SE. 2001. Can researchers conceal their scent from predators in artificial nest studies? *Wildlife Society Bulletin* 29:814-820.

Donovan TM, Jones PW, Annand EM, and Thompson III FR. 1997. Variation in local-scale edge effects: mechanisms and landscape context. *Ecology* 78:2064-2075 DOI:10.1890/0012-9 658(1997)078[2064:VILSEE]2.0.CO;2.

Dreher CE, Cummings ME, and Pröhl H. 2015. An analysis of predator selection to affect aposematic coloration in a poison frog species. *PLoS ONE* 10:e0134628 DOI:10.1371/ journal.pone.0130571.

Einarsen G, Hausner VH, Yoccoz NG, and Ims RA. 2008. Predation on artificial ground nests in birch forests fragmented by spruce plantations. *Ecoscience* 15:141-149 DOI:10.2980/15- 2-3025.

Erdős S, Báldi A, and Batáry PP. 2011. Relationship between grazing intensity, vegetation structure and survival of nests in semi-natural grasslands. *Acta Zoologica Academiae Scientiarum Hungaricae* 57:385-395.

Eriksson LM, Edenius L, Areskoug V, and Meritt Jr DA. 2001. Nest-predation at the edge: an experimental study contrasting two types of edges in the dry Chaco, Paraguay.  *Ecography* 24:742-750 DOI:10.1111/j.1600-0587.2001.tb00535.x.

Esler D, and Grand JB. 1993. Factors influencing depredation of artificial duck nests. *The Journal of Wildlife Management* 57:244-248 DOI:10.2307/3809420.

Farallo VR, and Forstner MRJ. 2012. Predation and the maintenance of color polymorphism in a habitat specialist squamate. *PLoS ONE* 7:e30316 DOI:10.1371/journal.pone.0030316.

Feeley KJ, and Terborgh JW. 2008. Direct versus indirect effects of habitat reduction on the loss of avian species from tropical forest fragments. *Animal Conservation* 11:353-360 DOI: 10.1111/j.1469-1795.2008.00182.x.

Fenske-Crawford TJ, and Niemi GJ. 1997. Predation of artificial ground nests at two types of edges in a forest-dominated landscape. *The Condor* 99:14-24 DOI:10.2307/1370220.

Finkbeiner SD, Briscoe AD, and Reed RD. 2012. The benefit of being a social butterfly: communal roosting deters predation. *Proceedings of the Royal Society of London B: Biological Sciences* 279:2769-2776 DOI:10.1098/rspb.2012.0203.

Finkbeiner SD, Briscoe AD, and Reed RD. 2014. Warning signals are seductive: relative contributions of color and pattern to predator avoidance and mate attraction in *Heliconius* butterflies. *Evolution* 68:3410-3420 DOI:10.1111/evo.12524.

Finkbeiner SD, Fishman DA, Osorio D, and Briscoe AD. 2017a. Ultraviolet and yellow reflectance but not fluorescence is important for visual discrimination of conspecifics by  *Heliconius erato*. *Journal of Experimental Biology* 220:1267-1276 DOI:10.1242/jeb. 153593.

Finkbeiner SD, Briscoe AD, and Mullen SP. 2017b. Complex dynamics underlie the evolution of imperfect wing pattern convergence in butterflies. *Evolution* 71:949-959 DOI:10.1111/ evo.13215.

Fitzpatrick BM, Shook K, and Izally R. 2009. Frequency-dependent selection by wild birds promotes polymorphism in model salamanders. *BMC Ecology* 9:12 DOI:10.1186/1472- 6785-9-12.

Flegeltaub M, Biro PA, and Beckmann C. 2017. Avian nest abandonment prior to laying—a strategy to minimize predation risk? *Journal of Ornithology* 158:1091-1098 DOI:10. 1007/s10336-017-1470-7.

França FGR, Braz VS, and Araújo AFB. 2017. Selective advantage conferred by resemblance of aposematic mimics to venomous model. *Biota Neotropica* 17:e20170338 DOI:10.1590/ 1676-0611-BN-2017-0338.

Francis CD, Ortega CP, Kennedy RI, and Nylander PJ. 2012. Are nest predators absent from noisy areas or unable to locate nests? *Ornithological Monographs* 74:101-110 DOI:10.15 25/om.2012.74.1.101.

Fraser FJ, and Whitehead PJ. 2005. Predation of artificial ground nests in Australian tropical savannas: inverse edge effects. *Wildlife Research* 32:313-319 DOI:10.1071/WR04021.

Fulton GR, and Ford HA. 2001. The pied currawong’s role in avian nest predation: a predator removal experiment. *Pacific Conservation Biology* 7:154-160 DOI:10.1071/PC010154.

Fulton GR, and Ford HA. 2003. Quail eggs, modelling clay eggs and small mammals in an Australian woodland. *Emu* 103:255-258 DOI:10.1071/MU02007.

Gabrey SW, Wilson BC, and Afton AD. 2002. Success of artificial bird nests in burned gulf coast chenier plain marshes. *The Southwestern Naturalist* 47:532-538 DOI:10.2307/3672 656.

Gardner JL. 1998. Experimental evidence for edge-related predation in a fragmented agricultural landscape. *Australian Journal of Ecology* 23:311-321 DOI:10.1111/j.1442-9993.1998.tb 00736.x.

George TL. 1987. Greater land bird densities on island vs. mainland: relation to nest predation level. *Ecology* 68:1393-1400 DOI:10.2307/1939223.

Gering JC, and Blair RB. 1999. Predation on artificial bird nests along an urban gradient: predatory risk or relaxation in urban environments? *Ecography* 22:532-541 DOI:10.1111/ j.1600-0587.1999.tb01283.x.

Gibbs JP. 1991. Avian nest predation in tropical wet forest: an experimental study. *Oikos* 60: 155-161 DOI:10.2307/3544861.

Gillis H, Gauffre B, Huot R, and Bretagnolle V. 2012. Vegetation height and egg coloration differentially affect predation rate and overheating risk: an experimental test mimicking a ground-nesting bird. *Canadian Journal of Zoology* 90:694-703 DOI:10.1139/Z2012-035.

Giroux MA, Trottier-Paquet M, Bêty J, Lamarre V, and Lecomte N. 2016. Is it safe to nest near bold neighbours? Spatial patterns in predation risk associated with the density of American golden-plover nest. *PeerJ* 4:e2193 DOI:10.7717/peerj.2193.

Githiru M, Lens L, and Cresswell W. 2005. Nest predation in a fragmented Afrotropical forest: evidence from natural and artificial nests. *Biological Conservation* 123:189-196 DOI:10. 1016/j.biocon.2004.11.006.

Göransson G, Karlsson J, Nilsson SG, and Ulfstrand S. 1975. Predation on birds’ nests in relation to antipredator aggression and nest density: an experimental study. *Oikos* 26:117- 120 DOI:10.2307/3543700.

Götmark F, and Åhlund M. 1984. Do field observers attract nest predators and influence nesting success of common eiders? *The Journal of Wildlife Management* 48:381-387 DOI:10.230 7/3801169.

Götmark F, Neergaard R, and Åhlund M. 1990. Predation of artificial and real arctic loon nests in Sweden. *The Journal of Wildlife Management* 54:429-432 DOI:10.2307/3809653.

Gottfried BM. 1978. An experimental analysis of the interrelationship between nest density and predation in old-field habitats. *The Wilson Bulletin* 90:643-646.

Gottfried BM, and Thompson CF. 1978. Experimental analysis of nest predation in an old-field habitat. *The Auk* 95:304-312.

Greenberg CH, Rossell Jr CR, and Johnson DB. 2002. Predation on artificial nests in hurricane- created gaps and adjacent forest of the southern Appalachians. *Journal of the North Carolina Academy of Science* 118:181-188.

Grieves LA, and Forbes S. 2012. Do sora nests protect red-winged blackbirds from marsh wren predation? *The Wilson Journal of Ornithology* 124:188-190 DOI:10.1676/1559-4491-12 4.1.188.

Grosse AM, Crawford BA, Maerz JC, Buhlmann KA, Norton T, Kaylor M, and Tuberville TD. 2015. Effects of vegetation structure and artificial nesting habitats on hatchling sex determination and nest survival of diamondback terrapins. *Journal of Fish and Wildlife Management* 6:19-28 DOI:10.3996/082014-JFWM-063.

Guimarães M, and Sawaya RJ. 2011. Pretending to be venomous: is a snake’s head shape a trustworthy signal to a predator? *Journal of Tropical Ecology* 27:437-439 DOI:10.1017/ S0266467411000095.

Guyn KL, and Clark RG. 1997. Cover characteristics and success of natural and artificial duck nests. *Journal of Field Ornithology* 68:33-41.

Haddad S, Desrochers A, and Savard JPL. 2000. Artificial nest predation in bogs: does peat harvest increase risk? *Ecoscience* 7:32-37 DOI:10.1080/11956860.2000.11682568.

Hamao S. 2005. Predation risk and nest-site characteristics of the black-browed reed warbler  *Acrocephalus bistrigiceps*: the role of plant strength. *Ornithological Science* 4:147-153 DOI:10.2326/osj.4.147.

Hamao S, Nishimatsu K, and Kamito T. 2009. Predation of bird nests by introduced Japanese weasel *Mustela itatsi* on an island. *Ornithological Science* 8:139-146 DOI:10.2326/osj.8. 139.

Hamilton AM, Freedman AH, and Franz R. 2002. Effects of deer feeders, habitat and sensory cues on predation rates on artificial turtle nests. *The American Midland Naturalist* 147: 123-134 DOI:10.1674/0003-0031(2002)147[0123:EODFHA]2.0.CO;2.

Hammond MC, and Forward WR. 1956. Experiments on causes of duck nest predation. *The Journal of Wildlife Management* 20:243-247 DOI:10.2307/3796957.

Hanmer HJ, Thomas RL, and Fellowes MDE. 2017. Provision of supplementary food for wild birds may increase the risk of local nest predation. *Ibis* 159:158-167 DOI:10.1111/ibi.12 432.

Hansson B, Bensch S, and Hasselquist D. 2000. Patterns of nest predation contribute to polygyny in the great reed warbler. *Ecology* 81:319-328 DOI:10.2307/177429.

Hanzelka J, and Reif J. 2015. Relative predation rate of artificial nests in the invasive black locust and semi-natural oak stands. *Sylvia* 51:63-73.

Harper GR, and Pfennig DW. 2007. Mimicry on the edge: why do mimics vary in resemblance to their model in different parts of their geographical range? *Proceedings of the Royal Society of London B: Biological Sciences* 274:1955-1961 DOI:10.1098/rspb.2007.0558.

Haskell DG. 1996. Do bright colors at nests incur a cost due to predation? *Evolutionary Ecology* 10:285-288.

Hausmann F, Catterall CP, and Piper SD. 2005. Effects of edge habitat and nest characteristics on depredation of artificial nests in fragmented Australian tropical rainforest. *Biodiversity and Conservation* 14:2331-2345 DOI:10.1007/s10531-004-1667-y.

Hegna RH, and Mappes J. 2014. Influences of geographic differentiation in the forewing warning signal of the wood tiger moth in Alaska. *Evolutionary Ecology* 28:1003-1017 DOI:10.1007/s10682-014-9734-7.

Hegna RH, Saporito RA, Gerow KG, and Donnelly MA. 2011. Contrasting colors of an aposematic poison frog do not affect predation. *Annales Zoologici Fennici* 48:29-38 DOI: 10.5735/086.048.0103.

Hegna RH, Saporito RA, Donnelly MA. 2013. Not all colors are equal: predation and color polytypism in the aposematic poison frog *Oophaga pumilio*. *Evolutionary Ecology* 27: 831-845 DOI:10.1007/s10682-012-9605-z.

Henry VG. 1969. Predation on dummy nests of ground-nesting birds in the southern Appalachians. *The Journal of Wildlife Management* 33:169-172 DOI:10.2307/3799666.

Hernández MA, Martín A, and Nogales M. 1999. Breeding success and predation on artificial nests of the endemic pigeons Bolle’s laurel pigeon *Columba bollii* and white-tailed laurel pigeon *Columba junoniae* in the laurel forest of Tenerife (Canary Islands). *Ibis* 141:52-59 DOI:10.1111/j.1474-919X.1999.tb04262.x.

Hernández F, Henke SE, Silvy NJ, and Rollins D. 2001. Comparison of success between actual northern bobwhite and wild turkey nests and simulated nests. *Wildlife Society Bulletin* 29: 1212-1218.

Herranz J, Yanes M, and Suárez F. 2002. Does photo-monitoring affect nest predation? *Journal of Field Ornithology* 73:97-101.

Hinman KE, Throop HL, Adams KL, Dake AJ, McLauchlan KK, McKone MJ. 1997. Predation by free-ranging birds on partial coral snake mimics: the importance of ring width and color. *Evolution* 51:1011–1014 DOI:10.1111/j.1558-5646.1997.tb03684.x.

Ho S, Schachat SR, Piel WH, and Monteiro A. 2016. Attack risk for butterflies changes with eyespot number and size. *Royal Society Open Science* 3:150614 DOI:10.1098/rsos. 150614.

Hodson EE, and Lehtinen RM. 2017. Diverse evidence for the decline of an adaptation in a coral snake mimic. *Evolutionary Biology* 44:401-410 DOI:10.1007/s11692-017-9418-7.

Hogrefe TC, Yahner RH, and Piergallini NH. 1998. Depredation of artificial ground nests in a suburban versus a rural landscape. *Journal of the Pennsylvania Academy of Science* 72:3- 6.

Hořák D, Sedláček O, Tószögyová A, Albrecht T, Ferenc M, Jelínek V, and Storch D. 2011. Geographic variation in avian clutch size and nest predation risk along a productivity gradient in South Africa. *Ostrich* 82:175-183 DOI:10.2989/00306525.2011.607863.

Huhta E, Eramo M, and Jokimäki J. 2015. Predation risk of artificial ground nests in forest stands, edges, clear-cuts, and forested corridors as an ecological indicator. In: Weber RP, eds. *Old-growth forests and coniferous forests: ecology habitat and conservation*. New York: Nova Publisher, 37-53.

Huhta E, Jokimäki J, and Helle P. 1998. Predation on artificial nests in a forest dominated landscape – the effects of nest type, patch size and edge structure. *Ecography* 21:464-471 DOI:10.1111/j.1600-0587.1998.tb00437.x.

Husak JF, Macedonia JM, Fox SF, and Sauceda RC. 2006. Predation cost of conspicuous male coloration in collared lizards (*Crotaphytus collaris*): an experimental test using clay- covered model lizards. *Ethology* 112:572-580 DOI:10.1111/j.1439-0310.2005.01189.x.

James RE, and Clout MN. 1996. Nesting success of New Zealand pigeons (*Hemiphaga novaeseelandiae*) in response to a rat (*Rattus rattus*) poisoning programme at Wenderholm Regional Park. *New Zealand Journal of Ecology* 20:45-51.

Janzen DH. 1978. Predation intensity on eggs on the ground in two Costa Rican forests. *The American Midland Naturalist* 100:467-470 DOI:10.2307/2424849.

Jedlikowski J, Brzeziński M, and Chibowski P. 2015. Habitat variables affecting nest predation rates at small ponds: a case study of the little crake *Porzana parva* and water rail *Rallus aquaticus*. *Bird Study* 62:190-201 DOI:10.1080/00063657.2015.1031080.

Ježková M, Svobodová J, and Kreisinger J. 2014. Dynamics of rodent abundance and ground- nest predation risks in forest habitats of central Europe: no evidence for the alternative prey hypothesis. *Folia Zoologica* 63:269-280 DOI:10.25225/fozo.v63.i4.a6.2014.

Jiang A, Jiang D, Goodale E, and Wen Y. 2017. Nest predation on birds that nest in rock cavities in a tropical limestone forest of southern China. *Global Ecology and Conservation* 10: 154-158 DOI:10.1016/j.gecco.2017.03.004.

Jobin B, and Picman J. 1997. Factors affecting predation on artificial nests in marshes. *The Journal of Wildlife Management* 61:792-800 DOI:10.2307/3802186.

Jobin B, and Picman J. 2002. Predation on artificial nests in upland habitats adjacent to freshwater marshes. *The American Midland Naturalist* 147:305-314 DOI:10.1674/0003- 0031(2002)147[0305:POANIU]2.0.CO;2.

Johnson MD, Adams TL, Branston TM, Clark RD, Crombie WB, Germann DL, Ringstad AMI, Langendorf H, and Moore JL. 2005. Variables influencing predation of artificial duck nests in northwest coastal California. *Transactions of the Western Section of the Wildlife Society* 41:11-20.

Jokimäki J, Kaisanlahti-Jokimäki ML, Sorace A, Fernández-Juricic E, Rodriguez-Prieto I, and Jimenez MD. 2005. Evaluation of the “safe nesting zone” hypothesis across an urban gradient: a multi-scale study. *Ecography* 28:59-70 DOI:10.1111/j.0906-7590.2005.0400 1.x.

Jones DD, Conner LM, Warren RJ, and Ware GO. 2002. The effect of supplemental prey and prescribed fire on success of artificial nests. *The Journal of Wildlife Management* 66: 1112-1117 DOI:10.2307/3802942.

Jones HP, Williamhenry III R, Howald GR, Tershy BR, and Croll DA. 2005. Predation of artificial Xantus’s murrelet (*Synthliboramphus hypoleucus scrippsi*) nests before and after black rat (*Rattus rattus*) eradication. *Environmental Conservation* 32:320-325 DOI:10.1017/S0376892906002608.

Jones DD, Conner LM, Warren RJ, and Ware GO. 2010. Effects of a supplemental food source and nest density on success of artificial ground nests. *Proceedings of the Annual Conference of the Southeastern Association of Fish and Wildlife Agencies* 64:56-60.

Kakazu S, Toledo LF, and Haddad CFB. 2010. Color polymorphism in *Leptodactylus fuscus*  (Anura, Leptodactylidae): a defensive strategy against predators? *Herpetology Notes* 3: 69-72.

Kelly JP. 1993. The effect of nest predation on habitat selection by dusky flycatchers in limber pine-juniper woodland. *The Condor* 95:83-93 DOI:10.2307/1369389.

Keyser AJ. 2002. Nest predation in fragmented forests: landscape matrix by distance from edge interactions. *The Wilson Bulletin* 114:186-191 DOI:10.1676/0043-5643(2002)114[0186: NPIFFL]2.0.CO;2.

Kikuchi DW, and Pfennig DW. 2010a. High model abundance may permit the gradual evolution of Batesian mimicry: an experimental test. *Proceedings of the Royal Society of London B: Biological Sciences* 277:1041–1048 DOI:10.1098/rspb.2009.2000.

Kikuchi DW, and Pfennig DW. 2010b. Predator cognition permits imperfect coral snake mimicry. *The American Naturalist* 176:830-834 DOI:10.1086/657041.

Kim MR. 2009. Habitat preference and nest predation risk in the blackbird (*Turdus merula*). *Journal of Ecology and Environment* 32:41-45 DOI:10.5141/JEFB.2009.32.1.041.

King RB. 1987 Color pattern polymorphism in the Lake Erie water snake, *Nerodia sipedon insularum*. *Evolution* 41:241-255 DOI:10.1111/j.1558-5646.1987.tb05794.x.

King DI, DeGraaf RM, and Griffin CR. 1998. Edge-related nest predation in clearcut and groupcut stands. *Conservation Biology* 12:1412-1415 DOI:10.1111/j.1523-1739.1998.97 199.x.

Klausen KB, Pedersen ÅØ, and Yoccoz NG. 2010. Prevalence of nest predators in a sub-Arctic ecosystem. *European Journal of Wildlife Research* 56:221-232 DOI:10.1007/s10344- 009-0304-1.

Kraemer AC, Serb JM, and Adams DC. 2016. Both novelty and conspicuousness influence selection by mammalian predators on the colour pattern of *Plethodon cinereus* (Urodela: Plethodontidae). *Biological Journal of the Linnean Society* 118:889-900 DOI:10.1111/ bij.12780.

Kross SM, McDonald PG, and Nelson XJ. 2013. New Zealand falcon nests suffer lower predation in agricultural habitat than in natural habitat. *Bird Conservation International* 23:512-519 DOI:10.1017/S0959270913000130.

Kuchta SR. 2005. Experimental support for aposematic coloration in the salamander *Ensatina eschscholtzii xanthoptica*: implications for mimicry of Pacific newts. *Copeia* 2005:265- 271 DOI:10.1643/CH-04-173R.

Kurucz K, Batáry P, Frank K, and Purger JJ. 2015. Effects of daily nest monitoring on predation rate – an artificial nest experiment. *North-western Journal of Zoology* 11:219-224.

Langen TA, Bolger DT, and Case TJ. 1991. Predation of artificial bird nests in chaparral fragments. *Oecologia* 86:395-401 DOI:10.1007/BF00317607.

Larivière S, and Messier F. 1998. Effect of density and nearest neighbours on simulated waterfowl nests: can predators recognize high-density nesting patches? *Oikos* 83:12-20 DOI:10.2307/3546541.

Larivière S, and Messier F. 2001. Temporal patterns of predation of duck nests in the Canadian prairies. *The American Midland Naturalist* 146:339-344 DOI:10.1674/0003-0031(2001) 146[0339:TPOPOD]2.0.CO;2.

Latif QS, Heath SK, and Rotenberry JT. 2012. How avian nest site selection responds to predation risk: testing an ‘adaptive peak hypothesis’. *Journal of Animal Ecology* 81:127- 138 DOI; 10.1111/j.1365-2656.2011.01895.x.

Laurance WF, Garesche J, and Payne CW. 1993. Avian nest predation in modified and natural habitats in tropical Queensland: an experimental study. *Wildlife Research* 20:711-723 DOI:10.1071/WR9930711.

Leader N, and Yom-Tov Y. 1998. The possible function of stone ramparts at the nest entrance of the blackstart. *Animal Behaviour* 56:207-217 DOI:10.1006/anbe.1998.0766.

Leighton PA, Horrocks JA, and Kramer DL. 2009. How depth alters detection and capture of buried prey: exploitation of sea turtle eggs by mongooses. *Behavioral Ecology* 20:1299- 1306 DOI:10.1093/beheco/arp139.

Leimgruber P, McShea WJ, and Rappole JH. 1994. Predation on artificial nests in large forest blocks. *The Journal of Wildlife Management* 58:254-260 DOI:10.2307/3809388.

Lewis KP, and Montevecchi WA. 1999. Predation on different-sized quail eggs in an artificial- nest study in western Newfoundland. *Canadian Journal of Zoology* 77:1170-1173 DOI: 10.1139/z99-076.

Lewis RM, Armstrong DP, Joy MK, Richard Y, Ravine D, Berggren Å, and Boulton RL. 2009. Using artificial nests to predict nest survival at reintroduction sites. *New Zealand Journal of Ecology* 33:40-51.

Li D, Sun X, Lloyd H, Que P, Liu Y, Wan D, and Zhang Z. 2015. Reed parrotbill nest predation by tidal mudflat crabs: evidence for an ecological trap? *Ecosphere* 6:20 DOI:10.1890/ ES14-00326.1.

Lindell C. 2000. Egg type influences predation rates in artificial nest experiment. *Journal of Field Ornithology* 71:16-21 DOI:10.1648/0273-8570-71.1.16.

Lindell CA, Cohen EB, and Fritz JS. 2004. Are daily mortality rates for real and artificial clutches comparable? *Ornitologia Neotropical* 15:201-208.

Lloyd P. 2007. Predator control, mesopredator release, and impacts on bird nesting success: a field test. *African Zoology* 42:180-186 DOI:10.3377/1562-7020(2007)42[180:PCMRAI 2.0.CO;2.

Loiselle BA, and Hoppes WG. 1983. Nest predation in insular and mainland lowland rainforest in Panama. *The Condor* 85:93-95.

Loman J, and Göransson G. 1978. Egg shell dumps and crow *Corvus cornix* predation on simulated birds’ nests. *Oikos* 30:461-466 DOI:10.2307/3543341.

López-Flores V, MacGregor-Fors I, and Schondube JE. 2009. Artificial nest predation along a Neotropical urban gradient. *Landscape and Urban Planning* 92:90-95 DOI:10.1016/j. landurbplan.2009.03.001.

Luck GW. 2003. Differences in the reproductive success and survival of the rufous treecreeper (*Climacteris rufa*) between a fragmented and unfragmented landscape. *Biological Conservation* 109:1-14 DOI:10.1016/S0006-3207(02)00085-X.

Luck GW, Possingham HP, and Paton DC. 1999. Bird responses at inherent and induced edges in the Murray Mallee, South Australia. 2. Nest predation as an edge effect. *Emu* 99:170- 175 DOI:10.1071/MU99020.

Ludwig M, Schlinkert H, Holzschuh A, Fischer C, Scherber C, Trnka A, Tscharntke T, and Batáry P. 2012. Landscape-moderated bird nest predation in hedges and forest edges.  *Acta Oecologica* 45:50-56 DOI:10.1016/j.actao.2012.08.008.

Luginbuhl JM, Marzluff JM, Bradley JE, Raphael MG, and Varland DE. 2001. Corvid survey techniques and the relationship between corvid relative abundance and nest predation. *Journal of Field Ornithology* 72:556-572 DOI:10.1648/0273-8570-72.4.556.

Lumpkin HA, Pearson SM, and Turner MG. 2012. Effects of climate and exurban development on nest predation and predator presence in the southern Appalachian Mountains (U.S.A.).  *Conservation Biology* 26:679-688 DOI:10.1111/j.1523-1739.2012.01851.x.

MacIvor LH, Melvin SM, and Griffin CR. 1990. Effects of research activity on piping plover nest predation. *The Journal of Wildlife Management* 54:443-447 DOI:10.2307/3809656.

Madsen T. 1987. Are juvenile grass snakes, *Natrix natrix*, aposematically coloured? *Oikos* 48: 265-267 DOI:10.2307/3565512.

Maier TJ, and DeGraaf RM. 2000. Predation on Japanese quail vs. house sparrow eggs in artificial nests: small eggs reveal small predators. *The Condor* 102:325-332 DOI:10.1650/ 0010-5422(2000)102[0325:POJQVH]2.0CO;2.

Maina GG, and Jackson WM. 2003. Effects of fragmentation on artificial nest predation in a tropical forest in Kenya. *Biological Conservation* 111:161-169 DOI:10.1016/S0006-3207 (02)00259-8.

Major RE. 1990. The effect of human observers on the intensity of nest predation. *Ibis* 132:608- 612 DOI:10.1111/j.1474-919X.1990.tb00285.x.

Major RE. 1991. Identification of nest predators by photography, dummy eggs, and adhesive tape. *The Auk* 108:190-195.

Major RE, Pyke GH, Christy MT, Gowing G, and Hill RS. 1994. Can nest predation explain the timing of the breeding season and the pattern of nest dispersion of New Holland honeyeaters? *Oikos* 69:364-372 DOI:10.2307/3545849.

Major RE, Gowing G, and Kendal CE. 1996. Nest predation in Australian urban environments and the role of the pied currawong, *Strepera graculina*. *Australian Journal of Ecology* 21:399-409 DOI:10.1111/j.1442-9993.1996.tb00626.x.

Major RE, Christie FJ, Gowing G, and Ivison TJ. 1999. Elevated rates of predation on artificial nests in linear strips of habitat. *Journal of Field Ornithology* 70:351-364.

Malt J, and Lank D. 2007. Temporal dynamics of edge effects on nest predation risk for the marbled murrelet. *Biological Conservation* 140:160-173 DOI:10.1016/j.biocon.2007.08. 011.

Malt JM, and Lank DB. 2009. Marbled murrelet nest predation risk in managed forest landscapes: dynamic fragmentation effects at multiple scales. *Ecological Applications* 19: 1274-1287 DOI:10.1890/08-0598.1,

Malzer I, and Helm B. 2015. The seasonal dynamics of artificial nest predation rates along edges in a mosaic managed reedbed. *PLoS ONE* 10:e0140247 DOI:10.1371/journal.pone.01402 47.

Mankin PC, and Warner RE. 1992. Vulnerability of ground nests to predation on an agricultural habitat island in east-central Illinois. *The American Midland Naturalist* 128:281-291 DOI:10.2307/2426462.

Marchand MN, Litvaitis JA, Maier TJ, DeGraaf RM. 2002. Use of artificial nests to investigate predation on freshwater turtle nests. *Wildlife Society Bulletin* 30:1092–1098 DOI:10.230 7/3784278.

Marini MA, Robinson SK, and Heske EJ. 1995. Edge effects on nest predation in the Shawnee National Forest, southern Illinois. *Biological Conservation* 74:203-213 DOI:10.1016/000 6-3207(95)00032-Y.

Marini MA, and Weale ME. 1997. Density- and frequency-dependent predation of artificial bird nests. *Biological Journal of the Linnean Society* 62:195-208 DOI:10.1006/bijl.1997.0146.

Marshall KLA, Philpot KE, and Stevens M. 2015. Conspicuous male coloration impairs survival against avian predators in Aegean wall lizards, *Podarcis erhardii*. *Ecology and Evolution* 5:4115-4131 DOI:10.1002/ece3.1650.

Martin TE. 1987. Artificial nest experiments: effects of nest appearance and type of predator.  *The Condor* 89:925-928 DOI:10.2307/1368547.

Martin J-L, and Joron M. 2003. Nest predation in forest birds: influence of predator type and predator’s habitat quality. *Oikos* 102:641-653 DOI:10.1034/j.1600-0706.2003.12040.x.

Marzluff JM, and Neatherlin E. 2006. Corvid response to human settlements and campgrounds: causes, consequences, and challenges for conservation. *Biological Conservation* 130:301- 314 DOI:10.1016/j.biocon.2005.12.026.

Marzluff JM, Raphael MG, and Sallabanks R. 2000. Understanding the effects of forest management on avian species. *Wildlife Society Bulletin* 28:1132-1143.

Masoero G, Maurino L, Rolando A, and Chamberlain D. 2016. The effect of treeline proximity on predation pressure: an experiment with artificial nests along elevational gradients in the European Alps. *Bird Study* 63:395-405 DOI:10.1080/00063657.2016.1214106.

Mason LC, Desmond MJ, Agudelo MS. 2005. Influence of grassland type, nest type, and shrub encroachment on predation of artificial nests in Chihuahuan Desert grasslands. *Western North American Naturalist* 65:196-201.

Matessi G, and Bogliani G. 1999. Effects of nest features and surrounding landscape on predation rates of artificial nests. *Bird Study* 46:184-194 DOI:10.1080/000636599094611 30.

Matthews A, Dickman CR, Major RE. 1999. The influence of fragment size and edge on nest predation in urban bushland. *Ecography* 22:349-356 DOI:10.1111/j.1600-0587.1999.tb00 572.x.

Mazgajski T, and Rejt Ł. 2005. Forest fragment size affects edge effect in nest predation. *Polish Journal of Ecology* 53:233-242.

McCann N, Haskell D, and Meyer MW. 2004. Capturing common loon nest predators on 35mm film. *The Passenger Pigeon* 66:351-361.

McElroy MT. 2016. Teasing apart crypsis and aposematism – evidence that disruptive coloration reduces predation on a noxious toad. *Biological Journal of the Linnean Society* 117:285– 294 DOI:10.1111/bij.12669.

McGuire A, and Kleindorfer S. 2007. Nesting success and apparent nest-adornment in diamond firetails (*Stagonopleura guttata*). *Emu* 107:44-51 DOI:10.1071/MU06031.

McKinnon L, and Bêty J. 2009. Effect of camera monitoring on survival rates of high-Arctic shorebird nests. *Journal of Field Ornithology* 80:280-288 DOI:10.1111/j.1557-9263. 2009.00231.x.

McLean CA, Moussalli A, and Stuart-Fox D. 2010. The predation cost of female resistance. *Behavioral Ecology* 21:861-867 DOI:10.1093/beheco/arq072.

McMillan DM, and Irschick DJ. 2010. Experimental test of predation and competition pressures on the green anole (*Anolis carolinensis*) in varying structural habitats. *Journal of Herpetology* 44:272-278 DOI:10.1670/08-196.1.

Medeiros R, Ramos JA, Pedro P, and Thomas RJ. 2012. Reproductive consequences of nest site selection by little terns breeding on sandy beaches. *Waterbirds* 35:512-524 DOI:10.1675/ 063.035.0402.

Melampy MN, Kershner EL, and Jones MA. 1999. Nest predation in suburban and rural woodlots of northern Ohio. *The American Midland Naturalist* 141:284-292 DOI:10.1674/ 0003-0031(1999)141[0284:NPISAR]2.0.CO;2.

Melo C, and Marini MA. Predação de ninhos artificiais em fragmentos de matas do Brasil central. *Ornitologia Neotropical* 8:7-14.

Melville HIAS, Conway WC, Morrison ML, Comer CE, and Hardin JB. 2014. Artificial nests identify possible nest predators of eastern wild turkeys. *Southeastern Naturalist* 13:80-91 DOI:10.1656/058.013.0106.

Merrill RM, Wallbank RWR, Bull V, Salazar PCA, Mallet J, Stevens M, and Jiggins CD. 2012. Disruptive ecological selection on a mating cue. *Proceedings of the Royal Society of London B: Biological Sciences* 279:4907-4913 DOI:10.1098/rspb.2012.1968.

Mĕstková L, Romportl D, Albrecht T, Chuman T, and Cervený J. 2012. The effect of landscape fragmentation and habitat variables on nest predation of artificial ground nest in the Bohemian Forest. *Silva Gabreta* 18:109-121.

Mezquida ET, and Marone L. 2002. Microhabitat structure and avian nest predation risk in an open Argentinean woodland: an experimental study. *Acta Oecologica* 23:313-320 DOI: 10.1016/S1146-609X(02)01160-8.

Mezquida ET, and Marone L. 2003. Are results of artificial nest experiments a valid indicator of success of natural nests? *The Wilson Bulletin* 115:270-276 DOI:10.1676/02-117.

Mezquida ET, Quse L, and Marone L. 2004. Artificial nest predation in natural and perturbed habitats of the central Monte Desert, Argentina. *Journal of Field Ornithology* 75:364-371 DOI:10.1648/0273-8570-75.4.364.

Michalski F, and Norris D. 2014. Artificial nest predation rates vary depending on visibility in the eastern Brazilian Amazon. *Acta Amazonica* 44:393-396 DOI:10.1590/1809-43922013 02553.

Miller JR, and Hobbs NT. 2000. Recreational trails, human activity, and nest predation in lowland riparian areas. *Landscape and Urban Planning* 50:227-236 DOI:10.1016/S0169- 2046(00)00091-8.

Mitrovich MJ, and Cotroneo RA. 2006. Use of plasticine replica snakes to elicit antipredator behavior in the California ground squirrel (*Spermophilus beecheyi*). *The Southwestern Naturalist* 51:263-267 DOI:10.1894/0038-4909(2006)51[263:UOPRST]2.0.CO;2.

Mochida K, Zhang WY, and Toda M. 2015. The function of body coloration of the hai coral snake *Sinomicrurus japonicus boettgeri*. *Zoological Studies* 54:33 DOI:10.1186/s40555- 015-0110-2.

Møller AP. 1987. Egg predation as a selective factor for nest design: an experiment. *Oikos* 50: 91-94 DOI:10.2307/3565404.

Møller AP. 1989. Nest site selection across field-woodland ecotones: the effect of nest predation.  *Oikos* 56:240-246 DOI:10.2307/3565342.

Møller AP. 1990. Nest predation selects for small nest size in the blackbird. *Oikos* 57:237-240 DOI:10.2307/3565945.

Morris G, and Conner LM. 2016. Effects of forest management practices, weather, and indices of nest predator abundance on nest predation: a 12-year artificial nest study. *Forest Ecology and Management* 366:23-31 DOI:10.1016/j.foreco.2016.02.006.

Némethová D, L’avrinčíková M, and Beňová M. 2004. Monitoring of predation on artificial nests with different number of artificial eggs. *Sylvia* 40:79-88.

Newton JL, and Heske EJ. 2001. Predation on artificial nests in small grassland patches in east- central Illinois. *The American Midland Naturalist* 145:29-38 DOI:10.1674/0003-0031(20 01)145[0029:POANIS]2.0.CO;2.

Nguyen LP, Abraham KF, and Nol E. 2006. Influence of arctic terns on survival of artificial and natural semipalmated plover nests. *Waterbirds* 29:100-104 DOI:10.1675/1524-4695(200 6)29[100:IOATOS]2.0.CO;2.

Niehaus AC, Heard SB, Hendrix SD, and Hillis SL. 2003. Measuring edge effects on nest predation in forest fragments: do finch and quail eggs tell different stories? *The American Midland Naturalist* 149:335-343 DOI:10.1674/0003-0031(2003)149[0335:MEEONP]2.0. CO;2.

Niemuth ND, and Boyce MS. 1995. Spatial and temporal patterns of predation of simulated sage grouse nests at high and low nest densities: an experimental study. *Canadian Journal of Zoology* 73:819-825 DOI:10.1139/z95-096.

Niemuth ND, and Boyce MS. 1997. Edge-related nest losses in Wisconsin pine barrens. *The Journal of Wildlife Management* 61:1234-1239 DOI:10.2307/3802122.

Nilsson SG, Björkman C, Forslund P, and Höglund J. 1985. Egg predation in forest bird communities on islands and mainland. *Oecologia* 66:511-515 DOI:10.1007/BF00379342.

Niskanen M, and Mappes J. 2005. Significance of the dorsal zigzag pattern of *Vipera latastei gaditana* against avian predators. *Journal of Animal Ecology* 74:1091-1101 DOI:10. 1111/j.1365-j.1365-2656.2005.01008.x.

Nokelainen O, Valkonen J, Lindstedt C, and Mappes J. 2014. Changes in predator community structure shifts the efficacy of two warning signals in Arctiid moths. *Journal of Animal Ecology* 83:598-605 DOI:10.1111/1365-2656.12169.

Noonan BP, and Comeault AA. 2008. The role of predator selection on polymorphic aposematic poison frogs. *Biology Letters* 5:51–54 DOI:10.1098/rsbl.2008.0586.

Norrdahl K, Suhonen J, Hemminki O, and Korpimäki E. 1995. Predator presence may benefit: kestrels protect curlew nests against nest predators. *Oecologia* 101:105-109 DOI:10.100 7/BF0032806.

Noske RA, Fischer S, and Brook BW. 2008. Artificial nest predation rates vary among habitats in the Australian monsoon tropics. *Ecological Research* 23:519-527 DOI:10.1007/s 11284-007-0403-y.

Nour N, Matthysen E, and Dhondt AA. 1993. Artificial nest predation and habitat fragmentation: different trends in bird and mammal predators. *Ecography* 16:111-116 DOI:10.1111/j.16 00-0587.1993.tb00063.x.

Ocampo D, and Londoño GA. 2014. Tropical montane birds have increased nesting success on small river islands. *The Auk* 132:1-10 DOI:10.1642/AUK-14-71.1.

Oja R, Zilmer K, and Valdmann H. 2014. Spatiotemporal effects of supplementary feeding of wild boar (*Sus scrofa*) on artificial ground nest depredation. *PLoS ONE* 10:e0135254 DOI:10.1371/journal.pone.0135254.

Oliveira CWS, Almeida GP, Paiva LV, and França LF. 2013. Predation on artificial nests in open habitats of central Brazil: effects of time and egg size. *Biota Neotropica* 13:142-146 DOI: 10.1590/S1676-06032013000100016.

Olson R, and Rohwer FC. 1998. Effects of human disturbance on success of artificial duck nests.  *The Journal of Wildlife Management* 62:1142-1146 DOI:10.2307/3802549.

O’Reilly P, and Hannon SJ. 1988. Predation of simulated willow ptarmigan nests: the influence of density and cover on spatial and temporal patterns of predation. *Canadian Journal of Zoology* 67:1263-1267 DOI:10.1139/z89-180.

Ortega YK, and Capen DE. 2002. Roads as edges: effects on birds in forested landscapes. *Forest Science* 48:381-390.

Ortega CP, Ortega JC, Rapp CA, and Backensto SA. 1998. Validating the use of artificial nests in predation experiments. *The Journal of Wildlife Management* 62:925-932 DOI:10.2307/ 3802544.

Padyšákova E, Šálek M, Poledník L, Sedláček F, and Albrecht T. 2009. Removal of American mink increases the success of simulated nests in linear habitat. *Wildlife Research* 36:225- 230 DOI:10.1071/WR08022.

Padyšákova E, Šálek M, Poledník L, Sedláček F, and Albrecht T. 2010. Predation on simulated duck nests in relation to nest density and landscape structure. *Wildlife Research* 37:597- 603 DOI:10.1071/WR10043.

Paemelaere EAD, Guyer C, and Dobson S. 2013. The role of microhabitat in predation on females with alternative dorsal patterns in a small Costa Rican anole (Squamata: Dactyloidae). *Revista De Biologia Tropical* 61:887-895.

Paluh DJ, Hantak MM, Saporito RA. 2014. A test of aposematism in the dendrobatid poison frog *Oophaga pumilio*: the importance of movement in clay model experiments. *Journal of Herpetology* 48:249–254 DOI:10.1670/13-027.

Paluh DJ, Kenison EK, and Saporito RA. 2015. Frog or fruit? The importance of color and shape to bird predators in clay model experiments. *Copeia* 103:58–63 DOI:10.1643/CE-13-126.

Pangau-Adam MZ, Waltert M, and Mühlenberg M. 2006. Nest predation risk on ground and shrub nests in forest margin areas of Sulawesi, Indonesia. *Biodiversity and Conservation* 15:4143-4158 DOI:10.1007/s10531-005-3370-z.

Pärt T, and Wretenberg J. 2002. Do artificial nests reveal relative nest predation risk for real nests? *Journal of Avian Biology* 33:39-46 DOI:10.1034/j.1600-048X.2002.330107.x.

Pasitschniak-Arts M, and Messier F. 1995. Predator identification at simulated waterfowl nests using inconspicuous hair catchers and wax-filled eggs. *Canadian Journal of Zoology* 73: 984-990 DOI:10.1139/z95-115.

Patterson L, Kalle R, and Downs C. 2016. Predation of artificial bird nests in suburban gardens of KwaZulu-Natal, South Africa. *Urban Ecosystems* 19:615-630 DOI:10.1007/s11252- 016-0526-4.

Pederson ÅØ, Yoccoz NG, and Ims RA. 2009. Spatial and temporal patterns of artificial nest predation in mountain birch forests fragmented by spruce plantations. *European Journal of Wildlife Research* 55:371-384 DOI:10.1007/s10344-009-0253-8.

Pehlak H, and Lõhmus A. 2008. An artificial nest experiment indicates equal nesting success of waders in coastal meadows and mires. *Ornis Fennica* 85:66-71.

Penloup A, Martin JL, Gory G, Burnstein D, and Bretagnolle V. 1997. Distribution and breeding success of pallid swifts, *Apus pallidus*, on Mediterranean islands: nest predation by the roof rat, *Rattus rattus*, and nest site quality. *Oikos* 80:78-88 DOI:10.2307/3546518.

Pescador M, and Peris S. 2007. Influence of roads on bird nest predation: an experimental study in the Iberian Peninsula. *Landscape and Urban Planning* 82:66-71 DOI:10.1016/j. landurbplan.2007.01.017.

Petit KE, Petit LJ, and Petit DR. 1989. Fecal sac removal: do the pattern and distance of dispersal affect the chance of nest predation? *The Condor* 91:479-482 DOI:10.2307/1368 331.

Pfennig DW, Harcombe WR, Pfennig KS. 2001. Frequency-dependent Batesian mimicry. *Nature* 410:323 DOI:10.1038/35066628.

Pfennig DW, Harper Jr GR, Brumo AF, Harcombe WR, and Pfennig KS. 2007. Population differences in predation on Batesian mimics in allopatry with their model: selection against mimics is strongest when they are common. *Behavioral Ecology and Sociobiology* 61:505-511 DOI:10.1007/s00265-006-0278-x.

Pfennig DW, Akcali CK, and Kikuchi DW. 2015. Batesian mimicry promotes pre- and post- mating isolation in a snake mimicry complex. *Evolution* 69:1085-1090 DOI:10.1111/evo. 12624.

Picman J. 1988. Experimental study of predation on eggs of ground-nesting birds: effects of habitat and nest distribution. *The Condor* 90:124-131 DOI:10.2307/1368441.

Picman J. 1992. Egg destruction by eastern meadowlarks. *The Wilson Bulletin* 104:520-525.

Picman J, Milks ML, and Leptich M. 1993. Patterns of predation on passerine nests in marshes: effects of water depth and distance from edge. *The Auk* 110:89-94 DOI:10.1093/auk/110. 1.89.

Picozzi N. 1975. Crow predation on marked nests. *The Journal of Wildlife Management* 39:151- 155 DOI:10.2307/3800478.

Pierre JP, Bears H, and Paszkowski CA. 2001. Effects of forest harvesting on nest predation in cavity-nesting waterfowl. *The Auk* 118:224-230 DOI:10.1642/0004-8038(2001)118[022 4:EOFHON]2.0.CO;2.

Piper SD, and Catterall CP. 2004. Effects of edge type and nest height on predation of artificial nests within subtropical Australian eucalypt forests. *Forest Ecology and Management* 203:361-372 DOI:10.1016/j.foreco.2004.08.005.

Piper SD, and Catterall CP. 2006. Is the conservation value of small urban remnants of eucalypt forest limited by increased levels of nest predation? *Emu* 106:119-125 DOI:10.1071/MU 05043.

Piper SD, Catterall CP, and Olsen M. 2002. Does adjacent land use affect predation of artificial shrub-nests near eucalypt forest edges? *Wildlife Research* 29:127-133 DOI:10.1071/WR0 1072.

Polak M. 2014. Protective nesting association between the barred warbler *Sylvia nisoria* and the red-backed shrike *Lanius collurio*: an experiment using artificial and natural nests. *Ecological Research* 29:949-957 DOI:10.1007/s11284-014-1183-9.

Posa MRC, Sodhi NS, and Koh LP. 2007. Predation on artificial nests and caterpillar models across a disturbance gradient in Subic Bay, Philippines. *Journal of Tropical Ecology* 23: 27-33 DOI:10.1017/S0266467406003671.

Preiẞler K, and Pröhl H. 2017. The effects of background coloration and dark spots on the risk of predation in poison frog models. *Evolutionary Ecology* 31:683-694 DOI:10.1007/s10 682-017-9903-6.

Prokop P, and Trnka A. 2011. Why do grebes cover their nests? Laboratory and field tests of two alternative hypotheses. *Journal of Ethology* 29:17-22 DOI:10.1007/s10164-010-0214-4.

Purger JJ, and Mészáros LA. 2006. Possible effects of nest predation on the breeding success of ferruginous ducks *Aythya nyroca*. *Bird Conservation International* 16:309-316 DOI:10. 1017/S0959270906000451.

Purger JJ, Mészáros LA, and Purger D. 2004. Ground nesting in recultivated forest habitats – a study with artificial nests. *Acta Ornithologica* 39:141-145 DOI:10.3161/068.039.0211.

Purger JJ, Csuka S, and Kurucz K. 2008. Predation survival of ground nesting birds in grass and wheat fields: experiment with plasticine eggs and artificial nests. *Polish Journal of Ecology* 56:481-486.

Purger JJ, Mužinić J, and Purger D. 2011. Artificial ground nest survival in two abandoned farmland habitats on Šolta Island (Croatia). *Avian Biology Research* 4:17-22 DOI:10. 3184/175815511X13000366671256.

Purger JJ, Kurucz K, Csuka SZ, and Batáry P. 2012a. Do different plasticine eggs in artificial nests influence nest survival? *Acta Zoologica Academiae Scientiarum Hungaricae* 58: 369-378.

Purger JJ, Kurucz K, Tóth Á, and Batáry P. 2012b. Coating plasticine eggs can eliminate the overestimation of predation on artificial ground nests. *Bird Study* 59:350-352 DOI:10. 1080/00063657.2012.684550.

Purger JJ, Mužinić J, and Purger D. 2012c. Survival chances of ground nests in a meadow habitat: a case study in Vrana Lake Nature Park (Mediterranean region, Croatia). *Polish Journal of Ecology* 60:207-212.

Purger JJ, Kletečki E, Trócsányi B, Mužinić J, Széles GL, and Lanszki J. 2015. Daily survival rates of eggs in artificial ground and shrub bird nests on small Adriatic islands. *Ardeola* 62:383-390 DOI:10.13157/arla.62.2.2015.383.

Rangen SA, Clark RG, and Hobson KA. 1999. Influence of nest-site vegetation and predator community on the success of artificial songbird nests. *Canadian Journal of Zoology* 77: 1676-1681 DOI:10.1139/z99-141.

Rangen SA, Clark RG, and Hobson KA. 2000.Visual and olfactory attributes of artificial nests.  *The Auk* 117:136-146 DOI:10.1642/0004-8038(2000)117[0136:VAOAOA]2.0.CO;2.

Raphael MG, Mack DE, Marzluff JM, and Luginbuhl JM. 2002. Effects of forest fragmentation on populations of the marbled murrelet. *Studies in Avian Biology* 25:221-235.

Rees JD, Webb JK, Crowther MS, and Letnic M. 2015. Carrion subsidies provided by fishermen increase predation of beach-nesting bird nests by facultative scavengers. *Animal Conservation* 18:44-49 DOI:10.1111/acv.12133.

Reino L, Porto M, Morgado R, Carvalho F, Mira A, and Beja P. Does afforestation increase bird nest predation risk in surrounding farmland? *Forest Ecology and Management* 2010: 1359-1366 DOI:10.1016/j.foreco.2010.07.032.

Reitsma L. 1992. Is nest predation density dependent? A test using artificial nests. *Canadian Journal of Zoology* 70:2498-2500 DOI:10.1139/z92-336.

Reitsma LR, Holmes RT, and Sherry TW. 1990. Effects of removal of red squirrels,  *Tamiasciurus hudsonicus*, and eastern chipmunks, *Tamias striatus*, on nest predation in a northern hardwood forest: an artificial nest experiment. *Oikos* 57:375-380 DOI:10.230 7/3565967.

Reitsma LR, and Whelan CJ. 2000. Does vertical partitioning of nest sites decrease nest predation? *The Auk* 117:409-415 DOI:10.2307/4089722.

Remeš V. 2005a. Birds and rodents destroy different nests: a study of blackcap *Sylvia atricapilla* using the removal of nest concealment. *Ibis* 147:213-215 DOI:10.1111/j.1474-919X.200 4.00339.x.

Remeš V. 2005b. Nest concealment and parental behaviour interact in affecting nest survival in the blackcap (*Sylvia atricapilla*): an experimental evaluation of the parental compensation hypothesis. *Behavioral Ecology and Sociobiology* 58:326-333 DOI:10.100 7/s00265-005-0910-1.

Richards-Zawacki CL, Yeager J, and Bart HPS. 2013. No evidence for differential survival or predation between sympatric color morphs of an aposematic poison frog. *Evolutionary Ecology* 27:783-795 DOI:10.1007/s10682-013-9636-0.

Ringelman KM, Eadie JM, and Ackerman JT. 2012. Density-dependent nest predation in waterfowl: the relative importance of nest density versus nest dispersion. *Oecologia* 169: 695-702 DOI:10.1007/s00442-011-2228-1.

Rivera-López A, and MacGregor-Fors I. 2016. Urban predation: a case study assessing artificial nest survival in a neotropical city. *Urban Ecosystems* 19:649-655 DOI:10.1007/s11252- 015-0523-z.

Rivers JW, Cable TT, and Pontius JS. 2003. Influence of nest concealment and distance to habitat edge on depredation rates of simulated grassland bird nests in southeast Kansas.  *Transactions of the Kansas Academy of Science* 106:40-47 DOI:10.1660/0022-8443 (2003)106[0040:IONCAD]2.0.CO;2.

Robel RJ, Hughes JP, Keane TD, and Kemp KE. 2003. Do artificial nests reveal meaningful patterns of predation in Kansas grasslands? *The Southwestern Naturalist* 48:460-464 DOI:10.1894/0038-4909(2003)048<0460:DANRMP>2.0.CO;2.

Robertson O, Maron M, Buckley Y, House A, and McAlpine C. 2014. An ecological paradox: more woodland predators and less artificial nest predation in landscapes colonized by noisy miners. *Austral Ecology* 39:255-266 DOI:10.1111/aec.12074.

Rojas B, Rautiala P, and Mappes J. 2014. Differential detectability of polymorphic warning signals under varying light environments. *Behavioural Processes* 109:164-172 DOI:10. 1016/j.beproc.2014.08.014.

Rollinson N, and Brooks RJ. 2007. Marking nests increases the frequency of nest depredation in a northern population of painted turtles (*Chrysemys picta*). *Journal of Herpetology* 41: 174-176 DOI:10.1670/0022-1511(2007)41[174:MNITFO]2.0.CO;2.

Roos S. 2002. Functional response, seasonal decline and landscape differences in nest predation risk. *Oecologia* 133:608-615 DOI:10.1007/s00442-002-1056-8.

Roos S, and Pärt T. 2004. Nest predators affect spatial dynamics of breeding red-backed shrikes (*Lanius collurio*). *Journal of Animal Ecology* 73:117-127 DOI:10.1111/j.1365-2656.200 4.00786.x.

Roper JJ. 1992. Nest predation experiments with quail eggs: too much to swallow? *Oikos* 65: 528-530 DOI:10.2307/3545570.

Roper JJ. 2000. Experimental analysis of nest-sites and nest predation for a neotropical bird: stuck between a rock and a hard place. *Ararajuba* 8:85-91.

Rose TA, and Banks PB. 2007. Impacts of black rats *Rattus rattus* across an urban/bushland interface at Sydney’s North Head. In: Lunney D, Eby P, Hutchings P, and Burgin S, eds. *Pest or Guest: the Zoology of Overabundance*. Sydney: Royal Zoological Society of New South Wales, 66-75.

Rudnicky TC, and Hunter Jr ML. 1993. Avian nest predation in clearcuts, forests, and edges in a forest-dominated landscape. *The Journal of Wildlife Management* 57:358-364 DOI:10.23 07/3809434.

Rutherford JL. 2010. Factors affecting predation on wood turtle (*Glyptemys insculpta*) nests in the upper peninsula of Michigan. M.S. Thesis, Northern Michigan University.

Ryder TB, Reitsma R, Evans B, and Marra PP. 2010. Quantifying avian nest survival along an urbanization gradient using citizen- and scientist-generated data. *Ecological Applications* 20:419-426 DOI:10.1890/09-0040.1.

Šálek M. 2004. The spatial pattern of the black-billed magpie, *Pica pica*, contribution to predation risk on dummy nests. *Folia Zoologica* 53:57-64.

Šálek M, Svobodová J, Bejček V, and Albrecht T. 2004. Predation on artificial nests in relation to the numbers of small mammals in the Krušné hory Mts, the Czech Republic. *Folia Zoologica* 53:312-318.

Salonen V, and Penttinen A. 1988. Factors affecting nest predation in the great crested grebe: field observations, experiments and their statistical analysis. *Ornis Fennica* 65:13-20.

Salvidio S, Palumbi G, Romano A, and Costa A. 2017. Safe caves and dangerous forests? Predation risk may contribute to salamander colonization of subterranean habitats. *The Science of Nature* 104:20 DOI:10.1007/s00114-017-1443-y.

Sánchez-Lafuente AM, Alcántara JM, and Romero M. 1998. Nest-site selection and nest predation in the purple swamphen. *Journal of Field Ornithology* 69:563-576.

Sánchez-Oliver JS, Rey Benayas JM, and Carrascal LM. 2014. Local habitat and landscape influence predation of bird nests on afforested Mediterranean cropland. *Acta Oecologica* 58:35-43 DOI:10.1016/j.actao.2014.05.001.

Santos T, and Telleria JL. 1991. Effects of leafing and position on nest predation in a Mediterranean fragmented forest. *The Wilson Bulletin* 103:676-682.

Santos MB, Oliveira MCLM, Gonçalves TP, Almeida FM, Loebmann D, and Tozetti AM. 2013. Does human influence on coastal grasslands habitats affect predation pressure on snakes? *Biota Neotropica* 13:365-370 DOI:10.1590/S1676-06032013000100039.

Saporito RA, Zuercher R, Roberts M, Gerow KG, Donnelly, MA. 2007. Experimental evidence for aposematism in the Dendrobatid poison frog *Oophaga pumilio*. *Copeia* 2007:1006– 1011 DOI:10.1643/0045-8511(2007)7[1006:EEFAIT]2.0.CO;2.

Saracco JF, and Collazo JA. 1999. Predation on artificial nests along three edge types in a North Carolina bottomland hardwood forest. *The Wilson Bulletin* 111:541-549.

Sato NJ, Morimoto G, Noske RA, and Ueda K. 2010. Nest form, colour, and nesting habitat affect predation rates of Australasian warblers (*Gerygone* spp.) in tropical mangroves. *Journal of the Yamashina Institute for Ornithology* 42:65-78 DOI:10.3312/jyio.42.65.

Sato CF, Wood JT, Schroder M, Green K, Osborne WS, Michael DR, and Lindenmayer DB. 2014. *Journal of Applied Ecology* 51:13-22 DOI:10.1111/1365-2664.12168.

Schiegg K, Eger M, and Pasinelli G. 2007. Nest predation in reed buntings *Emberiza schoeniclus*: an experimental study. *Ibis* 149:365-373 DOI:10.1111/j.1474-919X.2007.00 654.x.

Schmidt KA, Nelis LC, Briggs N, and Ostfeld RS. 2005. Invasive shrubs and songbird nesting success: effects of climate variability and predator abundance. *Ecological Applications* 15:258-265 DOI:10.1890/03-5325.

Schuetz JG. 2004. Common waxbills use carnivore scat to reduce the risk of nest predation. *Behavioral Ecology* 16:133-137 DOI:10.1093/beheco/arh139.

Schüttler E, Klenke R, McGehee S, Rozzi R, and Jax K. 2009. Vulnerability of ground-nesting waterbirds to predation by invasive American mink in the Cape Horn Biosphere Reserve, Chile. *Biological Conservation* 142:1450-1460 DOI:10.1016/j.biocon.2009.02.013.

Sedláček O, Mikeš M, Albrecht T, Reif J, and Hořák D. 2014. Evidence for an edge effect on avian nest predation in fragmented Afromontane forests in the Bamenda-Banso highlands, NW Cameroon. *Tropical Conservation Science* 7:720-732 DOI:10.1177/194 008291400700410.

Seibold S, Hempel A, Piehl S, Bässler C, Brandl R, Rösner S, and Müller J. 2013. Forest vegetation structure has more influence on predation risk of artificial ground nests than human activities. *Basic and Applied Ecology* 14:687-693 DOI:10.1016/j.baae.2013.09. 003.

Seitz LC, and Zegers DA. 1993. An experimental study of nest predation in adjacent deciduous, coniferous and successional habitats. *The Condor* 95:297-304 DOI:10.2307/1369352.

Selva N, Berezowska-Cnota T, and Elguero-Claramunt I. 2014. Unforeseen effects of supplementary feeding: ungulate baiting sites as hotspots for ground-nest predation. *PLoS ONE* 9:e90740 DOI:10.1371/journal.pone.0090740.

Seymoure BM, and Aiello A. 2015. Keeping the band together: evidence for false boundary disruptive coloration in a butterfly. *Journal of Evolutionary Biology* 28:1618-1624 DOI: 10.1111/jeb.12681.

Sieving KE. 1992. Nest predation and differential insular extinction among selected forest birds of central Panama. *Ecology* 73:2310-2328 DOI:10.2307/1941477.

Skagen SK, Stanley TR, and Dillon MB. 1999. Do mammalian nest predators follow human scent trails in the shortgrass prairie? *The Wilson Bulletin* 111:415-420.

Slagsvold T. 1989. Experiments on clutch size and nest size in passerine birds. *Oecologia* 80: 297-302 DOI:10.1007/BF00379030.

Small MF, and Hunter ML. 1988. Forest fragmentation and avian nest predation in forested landscapes. *Oecologia* 76:62-64 DOI:10.1007/BF00379601.

Smith PA, Gilchrist HG, and Smith JNM. 2007. Effects of nest habitat, food, and parental behavior on shorebird nest success. *The Condor* 109:15–31 DOI:10.1650/0010-5422(200 7)109[15:EONHFA]2.0.CO;2.

Smith DHV, Wilson DJ, Moller H, and Murphy EC. 2008. Using artificial nests to explore predation by introduced predators inhabiting alpine areas in New Zealand. *New Zealand Journal of Zoology* 35:119-128 DOI:10.1080/03014220809510109.

Smith HM, Dickman CR, and Banks PB. 2016. Nest predation by commensal rodents in urban bushland remnants. *PLoS ONE* 11:e0156180 DOI:10.1371/journal.pone.0156180.

Sodhi NS, Castelleta M, Loh WZ, Lee BPY-H, and Subaraj R. 2004. Tropical lowland rainforest birds on a highly urbanized island: monitoring, losses and lessons. In: Shaw WW, Harris LK, and VanDruff L, eds. *Proceedings of the 4^th^ International Symposium on Urban Wildlife Conservation*. Tucson: University of Arizona, 78-86.

Song SJ, and Hannon SJ. 1999. Predation in heterogeneous forests: a comparison at natural and anthropogenic edges. *Ecosciene* 6:521-530 DOI:10.1080/11956860.1999.11682550.

Sosa RA, and de Casenave JL. 2017. Edge effect on bird nest predation in the fragmented caldén (*Prosopis caldenia*) forest of central Argentina: an experimental analysis. *Ecological Research* 32:129-134 DOI:10.1007/s11284-016-1421-4.

Spanhove T, Lehouck V, and Lens L. 2009. Inverse edge effect on nest predation in a Kenyan forest fragment: an experimental case study. *Bird Conservation International* 19:367-378 DOI:10.1017/S0959270909008752.

Staus NL, and Mayer PM. 1999. Arthropods and predation of artificial nests in the Bahamas: implications for subtropical avifauna. *The Wilson Bulletin* 111:561-564.

Steffen JE. 2009. Perch-height specific predation on tropical lizard clay models: implications for habitat selection in mainland neotropical lizards. *Revista de Biologia Tropical* 57:859- 864.

Steventon JD, Ott PK, and MacKenzie KL. 1999. Effect of partial cutting on predation risk to artificial bird nests. *Canadian Journal of Forest Research* 29:1911-1915 DOI:10.1139/ x99-193.

Stirnemann RL, Potter MA, Butler D, and Minot EO. 2015. Compounding effects of habitat fragmentation and predation on bird nests. *Austral Ecology* 40:974-981 DOI:10.1111/aec. 12282.

Støen OG, Wegge P, Heid S, Hjeljord O, and Nellemann O. 2010. The effect of recreational homes on willow ptarmigan (*Lagopus lagopus*) in a mountain area of Norway. *European Journal of Wildlife Research* 56:789-795 DOI:10.1007/s10344-010-0378-9.

Storaas T. 1988. A comparison of losses in artificial and naturally occurring capercaillie nests. *The Journal of Wildlife Management* 52:123-126 DOI:10.2307/3801071.

Storch I. 1990. Habitat fragmentation, nest site selection, and nest predation risk in capercaillie. *Ornis Scandinavica* 22:213-217 DOI:10.2307/3676592.

Storch I, Woitke E, and Krieger S. 2005. Landscape-scale edge effect in predation risk in forest- farmland mosaics of central Europe. *Landscape Ecology* 20:927-940 DOI:10.1007/s 10980-005-7005-2.

Strickland J, Colbert P, and Janzen FJ. 2010. Experimental analysis of effects of markers and habitat structure on predation of turtle nests. *Journal of Herpetology* 44:467-470 DOI: 10.1670/08-323.1.

Stuart YE, Dappen N, and Losin N. 2012. Inferring predator behavior from attack rates on prey- replicas that differ in conspicuousness. *PLoS ONE* 7:e48497 DOI:10.1371/journal.pone. 0048497.

Stuart-Smith AK, and Hayes JP. 2003. Influence of residual tree density on predation of artificial and natural songbird nests. *Forest Ecology and Management* 183:159-176 DOI:10.1016/ S0378-1127(03)00104-X.

Sullivan BD, and Dinsmore JJ. 1990. Factors affecting egg predation by American crows. *The Journal of Wildlife Management* 54:433-437 DOI:10.2307/3809654.

Summers RW, Green RE, Proctor R, Dugan D, Lambie D, Moncrieff R, Moss R, and Baines D. 2004. An experimental study of the effects of predation on the breeding productivity of capercaillie and black grouse. *Journal of Applied Ecology* 41:513-525 DOI:10.1111/j.002 1-8901.2004.00891.x.

Suvorov P, Svobodová J, and Albrecht T. 2014. Habitat edges affect patterns of artificial nest predation along a wetland-meadow boundary. *Acta Oecologica* 59:91-96 DOI:10.1016/j. actao.2014.06.003.

Svobodová J, Albrecht T, and Šálek M. 2004. The relationship between predation risk and occurrence of black grouse (*Tetrao tetrix*) in a highly fragmented landscape: an experiment based on artificial nests. *Ecoscience* 11:421-427 DOI:10.1080/11956860.200 4.11682851.

Svobodová J, Šálek M, and Albrecht T. 2007. Roads do not increase predation on experimental nests in a highly fragmented forest landscape. *Folia Zoologica* 56:84-89.

Svobodová J, Koubová M, Mrštný L, Albrecht T, and Kreisinger J. 2012. Temporal variation in nest predation risk along habitat edges between grassland and secondary forest in central Europe. *European Journal of Wildlife Research* 58:315-323 DOI:10.1007/s10344-011- 0582-2.

Swanson L, Sanyaolu RA, Gnoske T, Whelan CJ, Lonsdorf EV, and Cordeiro NJ. 2012. Differential response of nest predators to the presence of a decoy parent in artificial nests. *Bird Study* 59:96-101 DOI:10.1080/00063657.2011.645799.

Taylor LNH, and Ford HA. 1998. Predation of artificial nests in a fragmented landscape on the New England Tablelands of New South Wales. *Wildlife Research* 25:587-594 DOI:10.10 71/WR98034.

Thompson III FR, and Burhans DE. 2004. Differences in predators of artificial and real songbird nests: evidence of bias in artificial nest studies. *Conservation Biology* 18:373-380 DOI: 10.1111/j.1523-1739.2004.00167.x.

Thompson RG, Warkentin IG, and Flemming SP. Response to logging by a limited but variable nest predator guild in the boreal forest. *Canadian Journal of Forest Research* 38:1974- 1982 DOI:10.1139/X08-049.

Thorington KK, and Bowman R. 2003. Predation rate on artificial nests increases with human housing density in suburban habitats. *Ecography* 26:188-196 DOI:10.1034/j.1600-0587.2 003.03351.x.

Thurber DK, McClain WR, and Whitmore RC. 1994. Indirect effects of gypsy moth defoliation on nest predation. *The Journal of Wildlife Management* 58:493-500 DOI:10.2307/380932 1.

Trnka A, and Prokop P. 2011. The use and function of snake skins in the nests of great reed warblers *Acrocephalus arundinaceus*. *Ibis* 153:627-630 DOI:10.1111/j.1474-919X.201 1.01124.x.

Trnka A, Prokop P, and Batáry P. 2008. Dummy birds in artificial nest studies: an experiment with red-backed shrike *Lanius collurio*. *Bird Study* 55:329-331 DOI:10.1080/0006365080 9461539.

Trnka A, Batáry P, and Prokop P. 2009. Interacting effects of vegetation structure and breeding patterns on the survival of great reed warbler *Acrocephalus arundinaceus* nests. *Ardea* 97:109-116 DOI:10.5253/078.097.0113.

Trnka, Prokop P, and Batáry P. 2010. Infanticide or interference: does the great reed warbler selectively destroy eggs? *Annales Zoologici Fennici* 47:272-277 DOI:10.5735/086.047.0 405.

Trnka A, Peterková V, and Grujbárová Z. 2011. Does reed bunting (*Emberiza schoeniclus*) predict the risk of nest predation when choosing a breeding territory? An experimental study. *Ornis Fennica* 88:179-184.

Trujillo G, and Ahumada JA. 2005. Artificial nest experiments in a fragmented neotropical cloud forest. *Ornithologia Neotropical* 16:53-63.

Väänänen VM. 2000. Predation risk associated with nesting in gull colonies by two *Aythya* species: observation and an experimental test. *Journal of Avian Biology* 31:31-35 DOI: 10.1034/j.1600-048X.2000.310105.x.

Valkama J, and Currie D. 1999. Low productivity of curlews *Numenius arquata* on farmland in southern Finland: causes and consequences. *Ornis Fennica* 76:65-70.

Valkama J, Currie D, and Korpimäki E. 1999. Differences in the intensity of nest predation in the curlew *Numenius arquata*: a consequences of land use and predator densities? *Ecoscience* 6:497-504 DOI:10.1080/11956860.1999.11682552.

Valkonen J, Niskanen M, Björklund M, and Mappes J. 2011. Disruption or aposematism? Significance of dorsal zigzag pattern of European vipers. *Evolutionary Ecology* 25:1047- 1063 DOI:10.1007/s10682-011-9463-0.

Vander Haegen WM, and DeGraaf RM. 1996a. Predation on artificial nests in forested riparian buffer strips. *The Journal of Wildlife Management* 60:542-550 DOI:10.2307/3802071.

Vander Haegen WM, and DeGraaf RM. 1996. Predation rates on artificial nests in an industrial forest landscape. *Forest Ecology and Management* 86:171-179 DOI:10.1016/S0378-1127 (96)03776-0.

Vander Haegen WM, Schroeder MA, and DeGraaf RM. 2002. Predation on real and artificial nests in shrubsteppe landscapes fragmented by agriculture. *The Condor* 104:496-506 DOI:10.1650/0010-5422(2002)104[0496:PORAAN]2.0.CO;2.

Vander Lee BA, Lutz RS, Hansen LA, and Mathews NE. 1999. Effects of supplemental prey, vegetation, and time on success of artificial nests. *The Journal of Wildlife Management* 63:1299-1305 DOI:10.2307/3802848.

VanderWerf EA. 2001. Rodent control decreases predation on artificial nests in O‘ahu ‘Elepaio habitat. *Journal of Field Ornithology* 72:448-457 DOI:10.1648/0273-8570-72.3.448.

Vergara PM, and Simonetti JA. 2003. Forest fragmentation and rhinocryptid nest predation in central Chile. *Acta Oecologica* 24:285-288 DOI:10.1016/j.actao.2003.09.006.

Vergara PM, and Simonetti JA. 2004. Does nest-site cover reduce nest predation for rhinocryptids? *Journal of Field Ornithology* 75:188-191 DOI:10.1648/0273-8570-75.2.1 88.

Vögeli M, Laiolo P, Serrano D, and Tella JL. 2011. Predation of experimental nests is linked to local population dynamics in a fragmented bird population. *Biology Letters* 7:954-957 DOI:10.1098/rsbl.2011.0241.

Warren KA, and Ryan MR. 1999. Do internal fire lanes affect nest depredation rates in praires? *Prairie Naturalist* 31:215-220.

Watson CM, Roelke CE, Pasichnyk PN, and Cox CL. 2012. The fitness consequences of the autotomous blue tail in lizards: an empirical test predator response using clay models.  *Zoology* 115:339-344 DOI:10.1016/j.zool.2012.04.001.

Watters ME, McLash TL, Aldridge CL, and Brigham RM. 2002. The effect of vegetation structure on predation of artificial greater sage-grouse nests. *Ecoscience* 9:314-319 DOI: 10.1080/11956860.2002.11682718.

Webb JK, and Whiting MJ. 2005. Why don’t small snakes bask? Juvenile broad-headed snakes trade thermal benefits for safety. *Oikos* 110:515-522 DOI:10.1111/j.0030-1299.2005.137 22.x.

Webb JK, and Whiting MJ. 2006. Does rock disturbance by superb lyrebirds (*Menura novaehollandiae*) influence habitat selection by juvenile snakes? *Austral Ecology* 31:58- 67 DOI:10.1111/j.1442-9993.2006.01543.x.

Wee JLQ, and Monteiro A. 2017. Yellow and the novel aposematic signal, red, protect *Delias* butterflies from predators. *PLoS ONE* 12:e0168243 DOI:10.1371/journal.pone.0168243.

Weidinger K. 2001. How well do predation rates on artificial nests estimate predation on natural passerine nests? *Ibis* 143:632-641 DOI:10.1111/j.1474-919X.2001.tb04891.x.

Weidinger K. 2004. Relative effects of nest size and site on the risk of predation in open nesting passerines. *Journal of Avian Biology* 35:515-523 DOI:10.1111/j.0908-8857.2004.03244. x.

Whyte BI, Didham RK, and Briskie JV. 2005. The effects of forest edge and nest height on nest predation in two differing New Zealand forest habitats. *New Zealand Natural Sciences* 30:19-34.

Wilcove DS. 1985. Nest predation in forest tracts and the decline of migratory songbirds.  *Ecology* 66:1211-1214 DOI:10.2307/1939174.

Willebrand T, and Marcström V. 1988. On the danger of using dummy nests to study predation.  *The Auk* 105:378-370 DOI:10.2307/4087508.

Willink B, García-Rodríguez A, Bolaños F, Pröhl H. 2014. The interplay between multiple predators and prey colour divergence. *Biological Journal of the Linnean Society* 113:580–589 DOI:10.1111/bij.12355.

Willson MF, De Santo TL, and Sieving KE. 2003. Red squirrels and predation risk to bird nests in northern forests. *Canadian Journal of Zoology* 81:1202-1208 DOI:10.1139/Z03-096.

Wilson GR, Brittingham MC, and Goodrich LJ. 1998. How well do artificial nests estimate success of real nests? *The Condor* 100:357-364 DOI:10.2307/1370277.

Wong TCM, Sodhi NS, and Turner IM. 1998. Artificial nest and seed predation experiments in tropical lowland rainforest remnants of Singapore. *Biological Conservation* 85:97-104 DOI:10.1016/S0006-3207(97)00145-6.

Yahner RH, and Cypher BL. 1987. Effects of nest location on depredation of artificial arboreal nests. *The Journal of Wildlife Management* 51:178-181 DOI:10.2307/3801651.

Yahner RH, and DeLong CA. 1992. Avian predation and parasitism on artificial nests and eggs in two fragmented landscapes. *The Wilson Bulletin* 104:162-168.

Yahner RH, and Mahan CG. 1996. Effects of egg type on depredation of artificial ground nests. *The Wilson Bulletin* 108:129-136.

Yahner RH, and Mahan CG. 1997. Effects of logging roads on depredation of artificial ground nests in a forested landscape. *Wildlife Society Bulletin* 25:158-162.

Yahner RH, and Mahan CG. 1999. Potential for predator learning of artificial arboreal nest locations. *The Wilson Bulletin* 111:536-540.

Yahner RH, and Morrell TE. 1991. Depredation of artificial avian nests in irrigated forests. *The Wilson Bulletin* 103:113-117.

Yahner RH, and Piergallini NH. 1998. Effects of microsite selection on predation of artificial ground nests. *The Wilson Bulletin* 110:439-442.

Yahner RH, and Scott DP. 1988. Effects of forest fragmentation on depredation of artificial nests. *The Journal of Wildlife Management* 52:158-161 DOI:10.2307/3801078.

Yahner RH, and Voytko RA. 1989. Effects of nest-site selection on depredation of artificial nests. *The Journal of Wildlife Management* 53:21-25 DOI:10.2307/3801298.

Yahner RH, and Wright AL. 1985. Depredation on artificial ground nests: effects of edge and plot age. *The Journal of Wildlife Management* 49:508-513 DOI:10.2307/3801563.

Yahner RH, Morrell TE, and Rachael JS. 1989. Effects of edge contrast on depredation of artificial avian nests. *The Journal of Wildlife Management* 53:1135-1138 DOI:10.2307/ 3809622.

Yahner RH, Mahan CG, and DeLong CA. 1993. Dynamics of depredation on artificial ground nests in habitat managed for ruffed grouse. *The Wilson Bulletin* 105:172-179.

Yang C, Møller AP, Ma Z, Li F, and Liang W. 2014. Intensive nest predation by crabs produces source-sink dynamics in hosts and parasites. *Journal of Ornithology* 155:219-223 DOI: 10.1007/s10336-013-1003-y.

Yang C, Wang J, and Liang W. 2016. Blocking of ultraviolet reflectance on bird eggs reduces nest predation by aerial predators. *Journal of Ornithology* 157:43-47 DOI:10.1007/s 10336-015-1243-0.

Zanette L. 2002. What do artificial nests tells us about nest predation? *Biological Conservation* 103:323-329 DOI:10.1016/S0006-3207(01)00143-4.

Zanette L, and Jenkins B. 2000. Nesting success and nest predators in forest fragments: a study using real and artificial nests. *The Auk* 117:445-454 DOI:10.1642/0004-8038(2000)117[0 445:NSANPI]2.0.CO;2.

Zuria I, Gates JE, and Castellanos I. 2007. Artificial nest predation in hedgerows and scrub forest in a human-dominated landscape of central Mexico. *Acta Oecologica* 31:158-167 DOI: 10.1016/j.actao.2006.07.005.
